# EphrinB2 regulates the emergence of a hemogenic endothelium from the aorta

**DOI:** 10.1038/srep27195

**Published:** 2016-06-02

**Authors:** Inn-Inn Chen, Arianna Caprioli, Hidetaka Ohnuki, Hyeongil Kwak, Catherine Porcher, Giovanna Tosato

**Affiliations:** 1 Laboratory of Cellular Oncology, Center for Cancer Research, National Cancer Institute, National Institutes of Health, Bethesda, MD 20892, USA; 2 MRC Molecular Haematology Unit, Weatherall Institute of Molecular Medicine, John Radcliffe Hospital, Oxford University, OX3 9DS Oxford, UK; 3 Marymount University, 2807 N Glebe Road, Arlington, VA 22207, USA

## Abstract

Adult-type intraembryonic hematopoiesis arises from specialized endothelial cells of the dorsal aorta (DA). Despite the critical importance of this specialized endothelium for establishment of hematopoietic stem cells and adult hematopoietic lineages, the mechanisms regulating its emergence are incompletely understood. We show that EphrinB2, a principal regulator of endothelial cell function, controls the development of endothelium producing adult-type hematopoiesis. The absence of EphrinB2 impairs DA-derived hematopoiesis. Transmembrane EphrinB2 and its EphB4 receptor interact in the emerging DA, which transiently harbors EphrinB2^+^ and EphB4^+^ endothelial cells, thereby providing an opportunity for bi-directional cell-to-cell signaling to control the emergence of the hemogenic endothelium. Embryonic Stem (ES) cell-derived EphrinB2^+^ cells are enriched with hemogenic endothelial precursors. EphrinB2 silencing impairs ES generation of hematopoietic cells but not generation of endothelial cells. The identification of EphrinB2 as an essential regulator of adult hematopoiesis provides important insight in the regulation of early hematopoietic commitment.

Formation of blood cells and vascular networks is essential for delivery of oxygen to the developing embryo and adult mammals. During mouse development, vascular endothelial cells and hematopoietic progenitor cells are anatomically proximal in the yolk sac (YS) where the earliest erythroid progenitors emerge from the blood islands around embryonic day (E)7.5^1^,^2^, and in the embryo proper where hematopoietic stem cells (HSCs) with full hematopoietic reconstitution potential arise predominantly from the ventral endothelium of the dorsal aorta (DA) around E10.5^1^,^3^,^4^,^5^. The discovery of DA endothelium as a direct source of adult-type hematopoietic cells, i.e. with self-renewing potential and capable of yielding all adult blood cell lineages^6^,^7^ and the identification of endothelial precursors with hemogenic activity in the YS^8^,^9^,^10^ provides evidence for a functional developmental relationship between endothelial and hematopoietic cells during development.

Advances in embryonic stem (ES) cell differentiation systems have confirmed a close developmental relationship between endothelial and hematopoietic precursors by showing that ES cells can be induced to differentiate *in vitro* into a bipotential “blast colony-forming” cell, which gives rise to endothelial and hematopoietic precursors^11^ and into a hemogenic endothelium, which produces blood cells^12^,^13^. Although considerable progress has been made in clarifying the transcriptional programs that orchestrate hematopoiesis, less is known about the steps responsible for the generation of hemogenic endothelial cells despite the critical importance of this process to the emergence of adult-type hematopoiesis from the DA^14^. This is attributable in part to limitations of current ES differentiation systems that have thus far failed to generate HSC from ES cells *in vitro*, making it difficult to correlate findings in the ES system with the complex spatio-temporal emergence of hematopoietic populations in the embryo.

The DA is the first artery to form in the embryo proper^15^. Endothelial cells arising from the splanchnopleural mesoderm organize to form two vessels running parallel to each other laterally to the neural tube, which start to fuse at the level of the 3rd somite around E9.5 forming the DA. The DA primordium precedes the emergence of the cardinal vein, the first vein to develop in the embryo proper. Definitive hematopoiesis (i.e. able to reconstitute the hematopoietic system of irradiated mice upon transplantation^16^) arises from the DA after the emergence of the cardinal vein, suggesting the possibility that an arterial identity distinguishing an artery from vein is required for initiation of DA-derived hematopoiesis. However, an arterial identity is an insufficient determinant for hematopoiesis from endothelium since the emergence of hematopoietic cells in the DA is restricted in time (beginning at E9.5 with the appearance of hematopoietic clusters^17^ and diminishing by E12) and in location, as it mostly involves the ventral floor of the DA in the trunk^1^,^18^.

EphrinB2 (EfnB2), a transmembrane signaling molecule expressed in arterial but not venous endothelial cells^19^,^20^,^21^, is detected at E8.25 in the DA before circulation is fully established and before the emergence of adult-type hematopoiesis, but it is not required for the development of the DA^20^,^22^,^23^. EfnB2 is also detected in the YS plexus at E8.5, after the appearance of primitive erythroid progenitors at about E7.5^22^. EfnB2 does not promote arterial characteristics of endothelial cells, a function attributed to Notch signaling^24^, but controls the repulsive sorting of arterial- and venous-fated endothelial cells into their respective arteries and veins acting through the EfnB2-specific EphB4 tyrosine kinase receptors, which are expressed in venous but not arterial endothelial cells^20^,^22^,^25^. At the 6–8 somite stage, the emergent EfnB2-positive DA transiently contains a subset of EfnB2-negative endothelial cells, many of which express the venous EphB4 marker^26^. By the 10–13 somite stage, the DA only contains EfnB2-expressing endothelial cells, attributed to relocation of the venous-fated EphB4-expressing cells into the cardinal vein^26^. Genetic experiments in mice have shown that the global or endothelial-restricted deficiency of EfnB2 leads to early lethality by E11.5 attributed to defective remodeling of vascular networks and to abnormal heart development^20^,^21^,^25^.

Although red blood cells are observed circulating in the EfnB2-null YS and embryo proper up to E9.5, likely reflecting YS hematopoiesis^20^,^21^,^22^,^25^, a comprehensive evaluation of potential roles of EfnB2 in developmental hematopoiesis have not been reported. We have combined mouse genetics and *ex vivo*/*in vitro* analyses to examine the effects of EfnB2 deficiency in YS and DA hematopoiesis, and have identified a novel role of EfnB2 as a regulator of hematopoiesis from the DA.

## Results

### EfnB2 is required for *ex vivo* hematopoiesis from aorta

**Figure 1:**
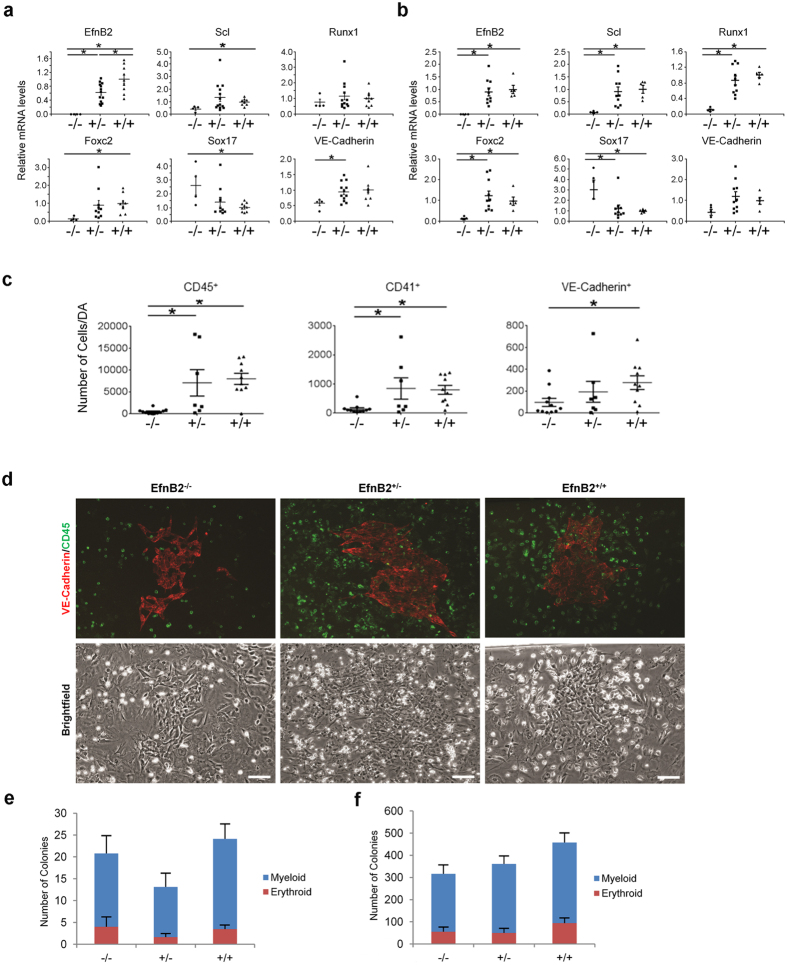
EfnB2 deficiency impairs *ex vivo* hematopoiesis from the DA but not YS. **(a,b)** Relative mRNA levels of the indicated genes in DAs resected from EfnB2^−/−^, EfnB2^+/−^ and EfnB2^+/+^ littermate embryos at E9.0–9.5 (**a)** 18–28 somite stage; EfnB2^−/−^: n = 4; EfnB2^+/−^: n = 13; EfnB2^+/+^: n = 8 or 9; four litters) and E10.0–10.5 (**b**) 20–36 somite stage; low somite counts reflect EfnB2^−/−^ embryo growth retardation; EfnB2^−/−^: n = 4; EfnB2^+/−^: n = 10; EfnB2^+/+^: n = 5; three litters) Individual data points (dot/square/triangle) are from individual DAs; mean (horizontal lines) ± SEM (error bars) are also shown. *P* values from unpaired Student *t*-test; *P < 0.05. **(c**) Number of CD45^+^, CD41^+^ and VE-Cadherin^+^ cells recovered from five-day OP9 co-culture of single-cell suspended E9.0–9.5 DAs (18–28 somite stage); EfnB2^−/−^ (n = 11), EfnB2^+/−^ (n = 7) and EfnB2^+/+^ (n = 10) littermates (four litters). Each data point (dot/square/triangle) represents the number of positive cells per resected aorta (1 embryo equivalent); mean (horizontal lines) ± SEM (error bars) are also shown. OP9 cells were excluded by forward/side scatter profiles. *P* values from unpaired Student *t*-test; *P < 0.05. **(d)** Immunofluorescent detection of VE-Cadherin (red) and CD45 (green) in representative Day 5 OP9 co-cultures of single-cell suspended DAs from E9.0–9.5 EfnB2^−/−^, EfnB2^+/−^ and EfnB2^+/+^ littermates. Corresponding brightfield images are shown. Scale bar: 100 μm. **(e,f)** Myeloid and erythroid colonies recovered after 9–12 days methylcellulose culture of single-cell suspended YSs from E7.5–8.5 (**e)** 0–12 somite stage; EfnB2^−/−^: n = 5; EfnB2^+/−^: n = 14; EfnB2^+/+^: n = 15) and E9.0–9.5 (**f**) 18–28 somite stage; EfnB2^−/−^: n = 6; EfnB2^+/−^: n = 9; EfnB2^+/+^: n = 13) littermates. Bar graphs show mean myeloid (blue) and erythroid (red) colonies/50 × 10^3^ cells) + SEM (error bars). *P* values (>0.05) are from unpaired Student *t*-test.

*EfnB2*-deficient mice die in utero by E11.5 due to cardiovascular defects attributed to defective myocardial trabeculation and remodeling of capillary networks, whereas the heterozygous mice are phenotypically indistinguishable from wild type as they are born alive and are fertile^20^,^22^. To study the impact of EfnB2 on the development of intraembryonic hematopoiesis, we dissected the DA^27^ from *EfnB2*^−/−^ mice and control *EfnB2*^+/−^ and *EfnB2*^+/+^ littermates at E9.0–9.5, just prior to or coincident with the emergence of hematopoietic clusters in the DA at E9.5^17^. At this time, the cardiovascular defects in EfnB2^−/−^ mice are unlikely to compromise hematopoiesis from the DA by reducing blood flow^28^ since circulation is not fully established^29^, the EfnB2^−/−^ embryos are viable as reflected by the presence of a beating heart and the EfnB2^−/−^ DAs are similar in appearance to control DAs, although somewhat enlarged in a proportion of mice^20^,^22^,^25^,^30^. We measured gene expression in individually-dissected DAs from mouse embryos at E9.0–9.5 and E10–10.5 with a focus on transcription factors linked to the regulation of hematopoiesis: *Scl/tal-1*, required for definitive-type hematopoiesis^31^ by conferring a hematopoietic fate to endothelial precursors^13^; *Runx1* required for endothelial-to-hematopoietic transition in the DA and production of all adult hematopoietic lineages^32^; *Foxc2*, a regulator of Notch signaling linked to arterial cell specification^33^; and *Sox17*, required for the formation or maintenance of a hemogenic endothelium^34^ and for repressing endothelial-to-hematopoietic transition^35^. The results from dissected aortas at E9.0–9.5 (Fig. 1a) and E10.0–10.5 (Fig. 1b) show that *EfnB2*^−/−^ DAs express the transcription factors *Scl/tal-1, Runx1* and *Foxc2* at abnormally low levels by E9.0–9.5 (*Scl* and *Foxc2*) and E10.0–10.5 (*Runx1*). Instead, consistent with *Sox17* role as a repressor of hematopoietic transcription factors^35^, *EfnB2*^−/−^ DAs express the transcription factor *Sox17* at a higher level than control DAs (Fig. 1a,b).

Notch signaling, which is essential for control of definitive-type hematopoiesis^36^,^37^,^38^, lies upstream of EfnB2^39^; we find no difference in relative mRNA levels of *Notch4, Dll4* and the Notch signaling mediator *Hey2* between *EfnB2*^−/−^ aortas and controls (Supplementary Fig. 1a,b). Similarly, we find no difference between *EfnB2*^−/−^ and control DAs in expression levels of *HoxA3* (Supplementary Fig. 1a), which represses hematopoiesis, in part through inhibiting *Runx1* expression^40^. Consistent with the *EfnB2*^−/−^ and WT DAs containing a similar number of CD31^+^ endothelial cells^30^, *VE-cadherin* expression was similar in *EfnB2*^−/−^, *EfnB2*^+/−^ and *EfnB2*^+/+^ DAs at E10–10.5 (Fig. 1a,b). Expectedly, *EfnB2* mRNA was not detected in DAs from *EfnB2*^−/−^ mice.

To ensure that the differences in gene expression (Fig. 1a,b) are not attributable to developmental retardation in the *EfnB2*^−/−^ embryos, we focused on embryos with a similar somite number (19–22 somites). We find that the *EfnB2*^−/−^ DAs express significantly lower relative mRNAs levels of *Scl, Runx1* and *Fox2*, and significantly higher relative mRNA levels of *Sox17* compared to the *EfnB2*^+/−^ or *EfnB2*^+/+^ DAs with a similar somite number (Supplementary Fig. 1c), indicating that altered gene expression is not attributable to delayed development in *EfnB2*^−/−^ DAs.

Since reduced expression of critical hematopoietic factors, particularly *Scl* and *Runx1*, suggested a defective hematopoietic program in *EfnB2*^−/−^ DAs, we assessed aortic hematopoiesis from E9.0–9.5 DAs *ex vivo*. Cells recovered from dissociation of individual littermate DAs were cultured for five days onto OP9 stromal cells in the presence of growth factors, as described^40^. We quantified by flow cytometry the output of hematopoietic cells (bearing surface CD45 or CD41) from each culture. We find that *EfnB2*^−/−^ DAs yielded significantly fewer CD45^+^ and CD41^+^ hematopoietic cells than *EfnB2*^+/−^ and *EfnB2*^+/+^ DAs (Fig. 1c, Supplementary Fig. 1d). We also find that the number of VE-cadherin^+^ cells recovered from *EfnB2*^−/−^ DAs was similar to the number of VE-cadherin^+^ cells recovered from EfnB2^+/−^ aortas, although lower than that from *EfnB2*^+/+^ aortas (Fig. 1c). A similar reduction of DA endothelial cells was observed previously raising the possibility that the loss of EphrinB2 may affect DA-derived endothelial cell proliferation or survival^30^. Fluorescence imaging confirmed the presence of numerous CD45^+^ cells (green) in five-day cultures of *EfnB2*^+/−^ and *EfnB2*^+/+^ aortic cell populations, which were rare in parallel cultures of EfnB2^−/−^ DA cell populations (Fig. 1d). This imaging also revealed similar colonies of adherent VE-cadherin^+^ cells (red) from *EfnB2*^+/+^, *EfnB2*^+/−^ and *EfnB2*^−/−^ DA cell cultures (Fig. 1d). These results provide evidence that EfnB2-deficient aortas are defective at generating hematopoietic cells *ex vivo*, but not at generating an endothelial monolayer.

Since these *ex vivo* results suggested that EfnB2 regulates hematopoiesis arising from the DA, we examined whether EfnB2 also regulates YS hematopoiesis, which begins at approximately E7.5, prior to EfnB2 detection in the YS vascular plexus at E8.5^22^. A first wave of YS hematopoiesis generates primitive erythroid cells, distinguished by their large size and embryonic globin expression, together with macrophages and megakaryocytes^41^. A second wave of YS hematopoiesis generates multipotential progenitors that differentiate into a variety of myeloid and erythroid cells, including erythroblasts containing only adult-type hemoglobin^41^. This second wave of YS hematopoiesis is detected by E9.5, prior to the development of HSC in the DA^41^. We utilized a methylcellulose medium enriched with the hematopoietic growth factors Stem Cell Factor, IL-3, IL-6, and Erythropoietin, which supports formation of YS-derived erythroid, granulocyte-macrophage from multipotential erythroid-myeloid progenitors (EMPs)^42^, but does not support the growth of single-cell suspended DAs from *EfnB2*^+/+^ and *EfnB2*^+/−^ embryos at E10–10.5 (not shown). We counted similar numbers of myeloid and erythroid colonies from YSs of *EfnB2*^−/−^, *EfnB2*^+/−^ and *EfnB2*^+/+^ embryos at E8.0–8.5 (Fig. 1e) and E9.0–9.5 (Fig. 1f), and observed a similar rapid rise in the content of colonies in the interval from E7.5–8.5 to E9.0–9.5, likely a reflection of the rapid rise of YS EMPs at this time^43^,^44^.

**Figure 2:**
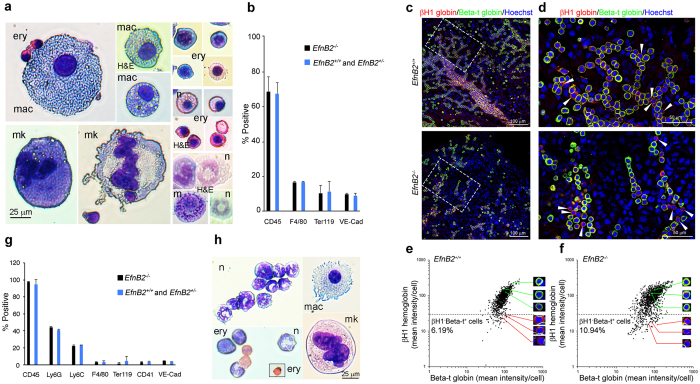
EphrinB2-deficient YSs generate a variety of erythroid and myeloid cells. **(a**) Morphology of EfnB2^−/−^ cytospun cells and **(b)** flow cytometric analysis of EfnB2^+/+^(n = 2) + EfnB2^+/−^ (n = 6) and EfnB2^−/−^ (n = 3) cells recovered from 5-day methylcellulose culture of single-cell suspended YSs at E9.5. Erythroid cells (ery) macrophages (mac), megakaryocytes (mk), neutrophils (n) and occasional mast cells (m) are identified morphologically; Wright’s stain or H&E where noted (**a**). Lineage-specific markers (**b**) show similar (all comparisons *P* values > 0.05) representation of hematopoietic (CD45^+^), erythroid cells (Ter119^+^), macrophages (F4/80^+^), erythroid (Ter119) and endothelial (VE-cadherin^+^) cells from individual cultures (colonies pooled) of E9.5 EfnB2^+/+^, EfnB2^+/−^ and EfnB2^−/−^ YSs. **(d–g)** Immunohistochemical detection of embryonic-type (βH1 globin, green) and adult-type (Beta-t globin, red) globins in nucleated (Hoechst^+^, blue) cells within EfnB2^+/+^ and EfnB2^−/−^ E9.0–9.5 YSs. The low magnification images (**d**) show areas (limited by a dotted line) magnified in (**e**) the arrowheads point to red-only cells that contain adult-type Beta-t globin only (**e**). Quantitation of nucleated cells containing adult-type Beta-t globin only (red) and nucleated cells containing embryonic-type (βH1^+^) globin alone (green) or with adult-type Beta-t globin (yellow) in EfnB2^+/+^ (n = 5) and EfnB2^−/−^ (n = 5) in E9.0–9.5 YSs (**f,g**). Each dot reflects the mean red and green fluorescence intensity in each cell; at least 200 cells were evaluated/YS; a total of 1164 (EfnB2^+/+^) and 1399 (EfnB2^−/−^) cells were measured; the dotted line limits background green fluorescence. The % cells containing adult-type Beta-t globin only (βH1^*−*^Beta-t^+^ cells) is shown. **(g)** Flow cytometric analysis of EfnB2^+/+^ (n = 2)+EfnB2^+/−^ (n = 3) and EfnB2^−/−^ (n = 3) cells recovered from 5-day OP9 culture of single-cell suspended YSs at E9.5 showing a similar distribution (all comparisons *P* values > 0.05) of lineage-specific markers, including CD11b^+^/Ly6G^+^ (Ly6G granulocytes), CD11b^+^/Ly6C^+^ (Ly6C monocytic) cells. **(h)** Morphology of EfnB2^−/−^ cytospun cells after staining with Wright’s stain. Error bars: standard deviations.

Cytospun cell suspensions of pooled colonies from E9.5 EfnB2^−/−^ YSs revealed representation of erythroid cells of different sizes with or without nuclei, large and small macrophages, megakaryocytes and occasional granulocytes (neutrophils and basophils) identified morphologically (Fig. 2a). Flow cytometry showed that CD45^+^ cells, Ter119^+^ erythroid cells, F4/80^+^ macrophages and VE-cadherin^+^ endothelial cells (Fig. 2b) were proportionally similar in pooled colony-derived cell suspensions from *EfnB2*^−/−^, *EfnB2*^+/−^ and *EfnB2*^+/+^ E9.5 YSs (Fig. 2b). Immunohistochemistry showed that *EfnB2*^+/+^ and *EfnB2*^−/−^ E9.0–9.5 YSs contain nucleated red cells with adult-type Beta-t globin protein (adult beta globins in C57BL mouse strains are: Beta-s and Beta-t) without embryonic-type βH1 globin protein, in addition to nucleated red cells containing embryonic-type βH1 globin protein alone or with adult-type Beta-t globin protein (Fig. 2c,d, Supplementary Fig. 2a–c). Quantitatively, erythroid cells with adult-type Beta-t globin protein only (no βH1 globin protein) were present in similar proportions in *EfnB2*^−/−^ and *EfnB2*^+/+^ YSs at E9.0–9.5 (Fig. 2e,f). When cultured for five days onto OP9 stromal cells (same conditions used for DA-derived cells^40^) *EfnB2*^−/−^, *EfnB2*^+/−^ and *EfnB2*^+/+^ E9.5 YS cells generated proportionally similar populations of CD45^+^, CD11b^+^/Ly6G^+^, CD11b^+^/Ly6C^+^, F4/80^+^, Ter119^+^, CD41^+^ and VE-cadherin^+^ cells (Fig. 2g). EfnB2^−/−^ YS-derived cells recovered from OP9 co-culture contained numerous granulocytes in addition to macrophages, megakaryocytes and erythroid cells (Fig. 2h). Together, there results demonstrate that EfnB2 is not critical for YS-derived hematopoiesis, including development of a second wave of hematopoiesis, and suggest that the requirement for EfnB2 can help distinguish the YS and DA hematopoietic programs.

### Proximity co-localization of EfnB2 and EphB4 in aortic endothelium

**Figure 3:**
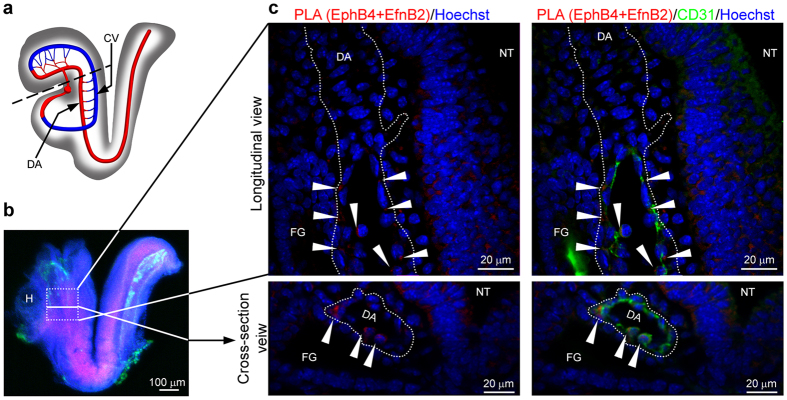
Association of EfnB2 and EphB4 expressed in neighboring endothelial cells of the developing DA. **(a)** Schematic lateral view of the DA in mouse embryo at E8.5; the dotted line indicates the location of head removal from embryo in test sample. CV: cardinal vein; DA: dorsal aorta. **(b)** Clarified embryo (E8.5, 8 somites) after removal of the head and side wall; CD31 immunostaining (green); Hoechst staining (blue); and PLA (EfnB2+EphB4, pink). The dotted box limits the longitudinal area magnified in the upper panels in c; the solid line within the dotted box indicates the plane of cross section for the area magnified in the bottom panels in c. H: heart. **(c)** PLA shows that EfnB2 is associated with EphB4 in the ventral floor of the DA from a WT embryo (8 somite stage); longitudinal slice shown in the upper panels; cross-section view is shown in the bottom panels. Red: EfnB2+EphB4 PLA signal; blue: Hoechst (DNA/nuclei); green: CD31 (endothelium). The DA is outlined by the dotted line. FG: foregut. NT: neural tube. The arrowheads point to PLA signal from proximity co-localization of EfnB2 and EphB4.

A functional role of EfnB2 in the selective emergence of a hemogenic endothelium from the DA likely requires signaling interaction with EphB4, the principal receptor for EfnB2. This requirement is reflected by the symmetrical vascular phenotypes of EphB4^−/−^ and EfnB2^−/−^ embryos^25^. EfnB2/EphB4 bidirectional signaling (EphB4-derived or EfnB2-derived) is initiated by interaction “in trans” of EfnB2 and EphB4 molecules anchored to the surface of neighboring cells^45^. Since recent studies have shown that a subset of EphB4^+^/EfnB2^*−*^ endothelial cells transiently co-habit the emerging DA with EfnB2^+^/EphB4^*−*^ endothelial cells at E8.0–8.5^26^, we examined whether this co-habitation provides an opportunity for EfnB2/EphB4 interaction during normal development. To this end, we performed proximity ligation assay (PLA) for EfnB2 and EphB4 in littermate *EfnB2*^+/+^ (Fig. 3) and *EfnB2*^−/−^ (Supplementary Fig. 3) embryos at E8.0–8.5. After embryo clarification to increase tissue transparency, we imaged at the confocal and obtained 3-D reconstructions of the aortas. PLA imaging in longitudinal and cross-section layers of the WT aorta at E8.5 (8-somite stage) showed that EfnB2 and EphB4 co-localize (<40 μm distance) in selected areas of the ventral floor of DA endothelium (Fig. 3). The ventral floor of the DA in the trunk has been recognized as the anatomical site of hematopoietic cell emergence from the DA^1^. Expectedly, no EfnB2 + EphB4 PLA signal was observed in the DA endothelium of an EfnB2^−/−^ littermate embryo (Supplementary Fig. 3), and EfnB2 + EphB4 PLA signal was strong in the WT neural tube (Fig. 3b), a developing structure where these molecules play critical signaling functions^46^,^47^. These results show that the temporally and spatially restricted co-habitation of EphB4^+^ and EfnB2^+^ endothelial cells in the developing DA provides an opportunity for EfnB2/EphB4 to interact and signal in selected endothelial cells of the developing DA thereby specifying a hemogenic endothelium from a non-hemogenic endothelium.

### EfnB2 requirement for hematopoiesis from ES-derived endothelium

**Figure 4:**
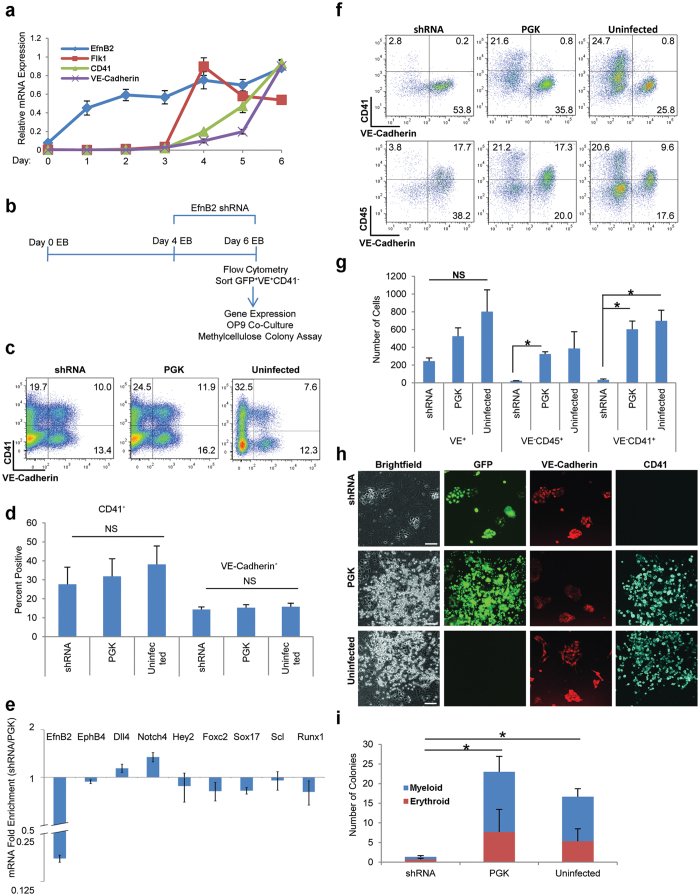
*EfnB2* silencing impairs hematopoiesis from ES-derived endothelium. **(a**) Kinetics of gene expression during ES/EB differentiation. Each GAPDH-normalized gene expression value was normalized across all time points (maximum expression = 1). Results reflect mean relative mRNA expression ± SEM (error bars); three independent experiments. **(b**) Schematic of experiment. Single-cell suspended Day 4 ES/EBs were re-aggregated at high density with EfnB2 shRNA/PGK/no lentivirus. After 48 hours, VE-Cadherin^+^CD41^*−*^ cells (GFP^+^ for shRNA/PGK) were sorted, analyzed and cultured (OP9 stroma or methylcellulose medium). VE: VE-Cadherin. **(c,d)** Surface VE-Cadherin and CD41 in shRNA, PGK and Uninfected ES/EB cells at differentiation Day 6 (GFP^+^ for shRNA/PGK). (**c**) Representative flow cytometry profiles (% cells in each quadrant). (**d**) Mean percent CD41^+^ and VE-Cadherin^+^ cells + SEM (error bars); three independent experiments. NS: non significant; *P* values (>0.05) from paired Student *t*-test. **(e)** Fold mRNA enrichment in GFP^+^VE^+^CD41^*−*^ cells (shRNA versus PGK); differentiation Day 6. Fold change: ratio of GAPDH-normalized value in the shRNA sample/corresponding GAPDH-normalized PGK value. Mean fold enrichment ± SEM (error bars); log_2_ scale bar: 10 μm; three independent experiments. **(f,g)** Surface VE-Cadherin, CD41 and CD45 in EfnB2 shRNA, PGK and uninfected cells after five-day culture on OP9 stroma; prior to OP9 co-culture, the VE-Cadherin^+^CD41^*−*^ cells were sorted on ES/EB differentiation Day 6 (GFP^+^ gate for shRNA and PGK). (**f**) Representative flow cytometry profiles (% cells in each quadrant); (**g**) Mean cell number recovered (from 25,000 cells re-plated) + SEM (error bars); three independent experiments. NS: non significant; *P value (<0.05) from paired Student *t*-test. **(h**) Immunofluorescent detection of VE-Cadherin (red), CD41 (blue) and GFP (green) after five-day OP9 co-culture of sorted VE-Cadherin^+^CD41^*−*^ cells (GFP^+^ shRNA & PGK samples); corresponding brightfield images (left quadrants). Scale bars: 200 μm **(i)** Myeloid (blue) and erythroid (red) colonies quantified after 9–12 days methylcellulose culture of sorted VE-Cadherin^+^CD41^*−*^ cells (GFP^+^ shRNA & PGK samples). Mean colony number (50,000 re-plated cells) + SEM (error bars) are shown; three independent experiments (paired Student *t*-test on total colony counts; *p < 0.05).

ES cells can generate hematopoietic cells *in vitro* through differentiation into a hemogenic endothelium, providing a source of hematopoietic cells from endothelium^12^,^13^. The ES-derived hemogenic endothelium could reflect either DA-derived, YS-derived endothelial hematopoiesis or a combination of both programs^9^,^10^,^48^. Since the experiments in Figs 1 and 2 indicated that EfnB2 is required for hematopoiesis from the DA but not the YS, we tested if EfnB2 is required for ES cell differentiation *in vitro* into a hemogenic endothelium. First, we examined the kinetics of EfnB2 expression during ES differentiation through formation of embryoid bodies (EB)^12^,^13^. We found that *EfnB2* is expressed as early as on Day 1 of ES/EB differentiation (Fig. 4a). Expression of *EfnB2* precedes expression of mesodermal *Flk1* (also known as *KDR/VEGFR2*), *VE-Cadherin* (also known as *CD144*) and hematopoietic *CD41* marker (also known as *integrin alpha-IIb*) that are established at Day 4 of ES/EB differentiation (Fig. 4a). Previous studies have shown that EfnB2 is expressed early in the avian embryo: at E2 the posterior part of individual avian somites expresses EfnB2, which is required and sufficient to promote ventral migration of somitic cells to form the primitive aorta^49^. Although EfnB2 is not required for the formation of the DA in the mouse, EfnB2 was detected in mouse embryos at somite stage 5–7^20^,^22^,^26^.

To evaluate the requirement for EfnB2 in ES differentiation into endothelial cells (VE-Cadherin^+^CD41^*−*^ cells in Day 6 ES/EB culture) that are functionally hemogenic, we successfully silenced EfnB2 (Fig. 4 and Supplementary Fig. 4) by dissociating and re-aggregating Day 4 EBs in the presence of a combination of two lentiviral shRNA constructs, based on previously successful EB transduction experiments^40^ (schematic of experiment Fig. 4b). Two days after infection (Day 6 ES/EB culture), cell viability and live cell yields were similar in control (uninfected and empty PGK vector-infected) and EfnB2-silenced cells (Supplementary Fig. 4a). At this time-point, *EfnB2* silencing did not significantly alter the proportion of cells with surface VE-Cadherin and CD41 in the unfractionated EB cell population (Fig. 4c,d). However, when we tested the sorted endothelial VE-Cadherin^+^CD41^*−*^ cells (sorting purities Supplementary Fig. 4b), we found that *EfnB2* silencing reduces *Foxc2* and *Runx1* mRNAs compared to controls (control PGK: Fig. 4e; uninfected control: Supplementary Fig. 4c). Although not uniformly, *Scl* mRNA was also reduced in this EfnB2-silenced population compared to controls (Fig. 4e, Supplementary Fig. 4c). Despite *Hey2, Dll4* and *Notch4* being expressed at similar levels in *EfnB2*^−/−^, *EfnB2*^+/−^ and *EfnB2*^+/+^ DAs and *Sox17* being expressed at higher levels in the *EfnB2*^−/−^ compared to *EfnB2*^+/−^ and *EfnB2*^+/+^ DAs (Fig. 1a,b), *Hey2* and *Sox17* mRNAs were generally lower in the EfnB2-silenced VE-Cadherin^+^CD41^*−*^ sorted cells compared to controls whereas *Dll4* and *Notch4* mRNAs were somewhat higher in EfnB2-silenced VE-Cadherin^+^CD41^*−*^ sorted cells compared to controls (Fig. 4e, Supplementary Fig. 4c). These differences in gene expression distinguishing EfnB2^−/−^ DA and EfnB2-silenced ES-derived endothelium likely reflect inherent limitations of the ES culture system.

The EfnB2-silenced and control VE-Cadherin^+^CD41^*−*^ cells were cultured for five days on OP9 stroma to generate hematopoietic cells (schematic of experiment Fig. 4b). Under these conditions, the EfnB2-silenced VE-Cadherin^+^CD41^*−*^ cells were less hemogenic compared to both PGK-infected and uninfected controls (Fig. 4f,g): the percentage of VE-Cadherin^*−*^CD41^+^ was reduced by ten-fold and the percentage of VE-Cadherin^*−*^CD45^+^ was reduced by six-fold in the shRNA cells compared to PGK controls (Fig. 4f). The double-positive VE-Cadherin^+^CD45^+^ cell population, probably reflecting endothelial CD45 expression^50^, were similarly represented in the shRNA cells compared to controls (Fig. 4f). Averaged over three independent experiments, the yield of VE-Cadherin^*−*^CD41^+^ cells was eighteen-fold lower and the number of VE-Cadherin^*−*^CD45^+^ hematopoietic cells was fifteen-fold lower in OP9 co-cultures of EfnB2-silenced cells compared to PGK control (Fig. 4g). The overall output of VE-Cadherin^+^ endothelial cells from EfnB2-silenced VE-Cadherin^+^CD41^*−*^ cells was not significantly impaired although some reduction was present (Fig. 4g). Imaging confirmed that the EfnB2-silenced (GFP^+^/green) VE-Cadherin^+^CD41^*−*^ population generates virtually no VE-Cadherin^*−*^CD41^+^ hematopoietic cells (blue), but produces similar colonies of VE-Cadherin^+^ (red) endothelial cells to those identified in the control cultures where EfnB2 is not silenced (Fig. 4h, Supplementary Fig. 5). These results suggest that EfnB2 is required for generation of a hemogenic endothelium from ES cells (through Day 6 ES/EB-derived VE-Cadherin^+^CD41^*−*^ cells) but not for generating or sustaining an ES-derived endothelium. This conclusion is strengthened by experiments in which EfnB2 was silenced at the outset of differentiation (from Day 0 ES/EB culture), and culture continued for five days before re-plating the VE-Cadherin^+^CD41^*−*^ cells onto OP9 stroma (Supplementary Fig. 6a,b). Consistent with the results shown in Fig. 4, *EfnB2* silencing at this earlier time-point reduced the output of CD45^+^ and CD41^+^ cells from ES cells (Supplementary Fig. 6c,d).

We next assayed the effect of *EfnB2* silencing on ES generation of hematopoietic colonies. In these experiments, the EfnB2-silenced and control VE-Cadherin^+^CD41^*−*^ cells from day 6 ES/EB were dissociated and single cells were plated in methylcellulose medium in the presence of Stem Cell Factor, IL-3, IL-6 and Erythropoietin. After 9 to 12-day culture, the EfnB2-silenced cells generated significantly fewer myeloid and erythroid colonies compared to controls (Fig. 4i). There are different explanations for the reduction in the number of colonies induced by the silencing of EfnB2 in this system. It is possible that this ES/EB differentiation system mainly reflects the presence of a DA-like hematopoietic progenitor population, and that EfnB2 silencing severely impairs the hematopoietic output from this DA-like precursor. Alternatively, this ES/EB differentiation system may reflect the presence of both DA-like and YS-like precursors, in which case EfnB2 silencing would impair the hematopoietic output from both precursors. Irrespective of the explanation, these results show that the silencing of *EfnB2* in ES/EB impairs ES hematopoietic differentiation into a hemogenic endothelium and this outcome resembles the impaired hematopoietic output displayed by the *EfnB2*^−/−^ aortas in *ex vivo* culture (Fig. 1d).

### EfnB2 is dispensable for hematopoiesis in Blast Colony-Forming Cell Culture

**Figure 5:**
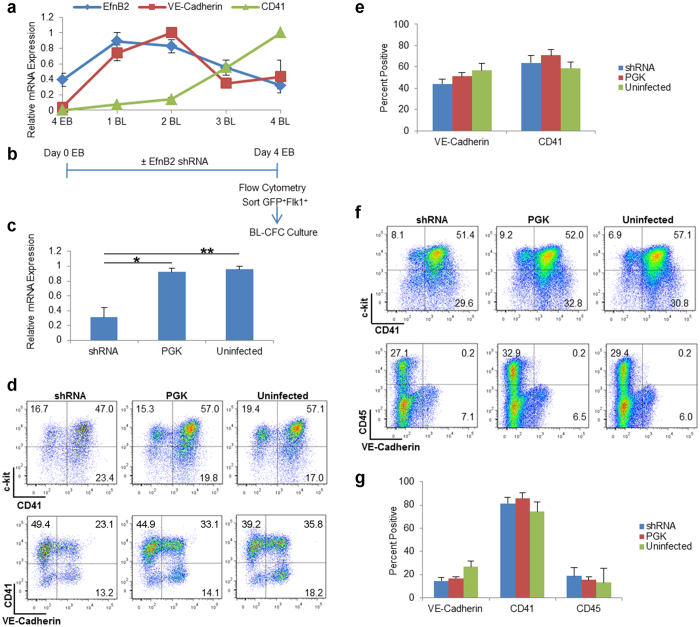
EfnB2 is not necessary for hematopoietic differentiation in Blast Colony-Forming Cell culture. **(a)** Kinetics of gene expression during Blast Colony-Forming Cell (BL-CFC) culture in Flk1^+^ cells sorted from Day 4 ES/EBs. Gene expression was evaluated in the Flk1^+^ cells sorted at Day 4 (4 EB) and at the indicated time-points during BL-CFC culture (1BL-4BL). Each GAPDH-normalized mRNA expression level was normalized across all time points (maximum expression = 1). Data points: mean relative mRNA expression ± SEM (error bars); three independent experiments. **(b)** Schematic of experiment. Day 0 ES/EBs were infected with shRNA or PGK, or left uninfected. At Day 4 of differentiation, Flk1^+^ cells (GFP^+^ for shRNA and PGK samples) were sorted and transferred to BL-CFC culture. **(c)** Relative *EfnB2* mRNA levels in Flk1^+^ (GFP^+^ for shRNA and PGK samples) cells sorted from shRNA/PGK/Uninfected ES/EBs on Day 4 of differentiation. Mean + SEM (error bars); three independent experiments (paired Student *t*-test; *p < 0.05, **p < 0.01). **(d–f)** Surface c-kit^+^, CD41^+^ and VE-Cadherin^+^ cells in Day 2 (**d,e**) and Day 4 (**f,g**) BL-CFC cultures of Flk1^+^ cells (GFP^+^ for shRNA and PGK samples) sorted on Day 4 of ES/EB differentiation. (**d,f**) Representative flow cytometry profiles (% cells in each quadrant indicated) (**e,g**) Mean percentage positive + SEM (error bars); three independent experiments; blue bars: shRNA-infected cells; red bars: PGK control infected cells; green bars: uninfected cells.

Previous studies have provided evidence for the existence of a bipotential precursor for hematopoietic and endothelial cells, referred to as “hemangioblast” in Flk1^+^ ES cells undergoing differentiation in methylcellulose-based blast colony forming cell (BL-CFC) culture^11^,^13^,^51^. A similar bipotential precursor capable of generating endothelial and hematopoietic cells was identified, if rarely, in the mouse embryo^52^. Given that EfnB2 mRNA is expressed in Flk1^+^ cells differentiated from ES cells at day 4 and continues to be expressed during subsequent BL-CFC culture (Fig. 5a), we tested if EfnB2 is required for hematopoietic differentiation from “hemangioblast” precursors in BL-CFC culture. Therefore, we silenced *EfnB2* at the outset of EB differentiation, sorted the silenced GFP^+^Flk1^+^ cells at Day 4 and cultured them into BL-CFC culture (schematic in Fig. 5b). The yield of Flk1^+^ cells at the time of sorting (Day 4 of ES/EB differentiation) was consistently, though insignificantly reduced by *EfnB2* silencing (Supplementary Table 1). Expectedly, *EfnB2* expression was reduced in the silenced GFP^+^Flk1^+^ cells sorted from Day 4 ES/EBs (Fig. 5c). Surprisingly, however, endothelial and hematopoietic differentiation proceeded normally from the EfnB2-silenced Flk1^+^ cells, as reflected by the normal distribution of VE-Cadherin^*−*^CD41^+^ and VE-Cadherin^*−*^CD45^+^ hematopoietic cells, and VE-Cadherin^+^ endothelial cells at Day 2 (Fig. 5d,e) and Day 4 (Fig. 5f,g) of BL-CFC differentiation. These results were reproduced when *EfnB2* was silenced later in ES/EB differentiation (Day 4 ES/EB) to avoid reduction of the Flk1^+^ cell population following early (Day 0 ES/EB) *EfnB2* silencing (Supplementary Fig. 7). Since the BL-CFC generates smooth muscle cells, in addition to endothelial and blood cells, and may thus sustain ES-derived mesodermal precursors with a broader differentiation potential than those generated in the OP9 system^53^, it is possible that the BL-CFC reflects an *in vitro* read out of primitive streak precursors of the YS hemogenic endothelium^10^. Altogether, these results provide evidence that EfnB2 is not required for hematopoietic differentiation from sorted Flk1^+^ cells in BL-CFC culture, mirroring the unperturbed hematopoietic colony formation from *EfnB2*^−/−^ YS.

### EfnB2 enriches for hemogenic cells in ES culture

**Figure 6:**
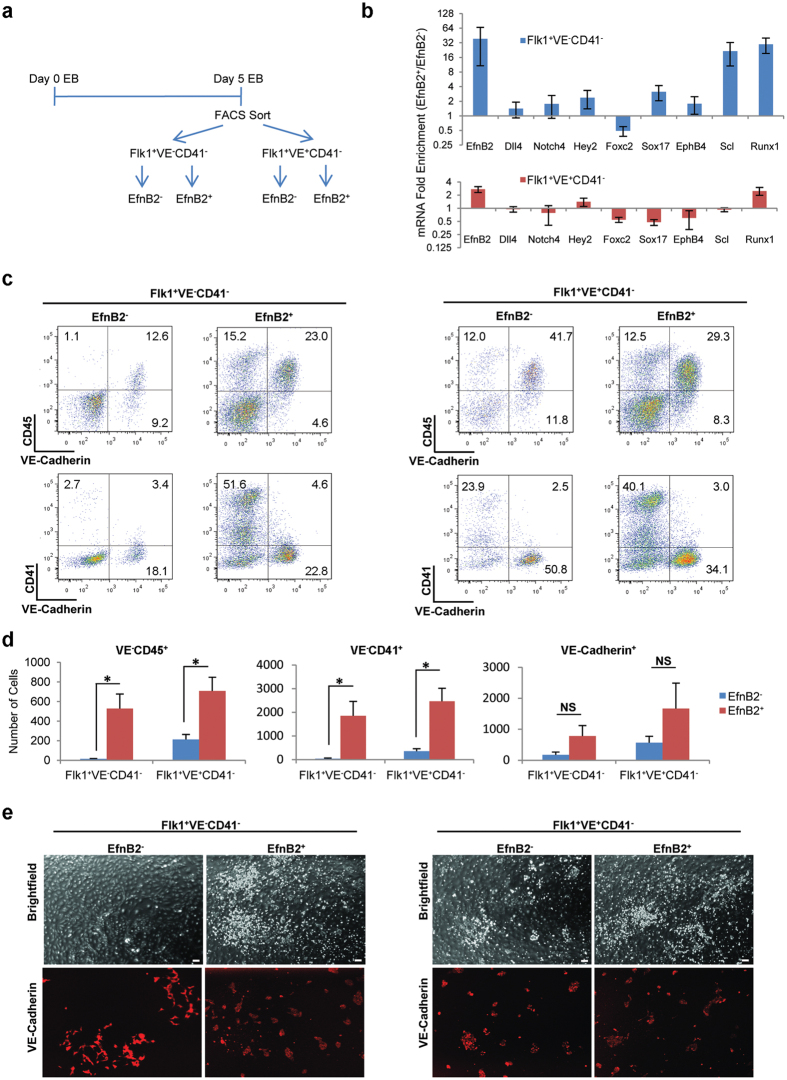
Endogenous EfnB2 expression marks ES cells with enhanced hematopoietic potential. **(a**) Schematic of experiment. EfnB2^+^ and EfnB2^*−*^ cells within the Flk1^+^VE-Cadherin^*−*^CD41^*−*^ and Flk1^+^VE-Cadherin^+^CD41^*−*^ populations were sorted from ES/EBs at Day 5 of differentiation. Cells were analyzed immediately and after five-day culture on OP9 stroma. **(b)** Selected mRNAs enrichment in sorted populations of EfnB2^+^ versus EfnB2^*−*^ cells from Day 5 ES/EB differentiation. Blue bars: Flk1^+^VE-Cadherin^*−*^CD41^*−*^; red bars: and Flk1^+^VE-Cadherin^+^CD41^*−*^. Fold enrichment is calculated as the ratio of GAPDH-normalized values in the EfnB2^+^ population divided by the corresponding EfnB2^*−*^ population. Mean fold enrichment ± SEM (error bars); log_2_ scale; three independent experiments. **(c,d)** Recovery of VE-Cadherin^*−*^CD45^+^, VE-Cadherin^*−*^CD41^+^, and VE-Cadherin^+^ cells from five-day OP9 co-culture of EfnB2^+^ and EfnB2^*−*^ cells (Flk1^+^VE-Cadherin^*−*^CD41^*−*^ and Flk1^+^VE-Cadherin^+^CD41^*−*^ populations). (**c**) Representative cell surface profiles; EfnB2^+^ and EfnB2^*−*^ cells of Flk1^+^VE-Cadherin^*−*^CD41^*−*^ (left) and Flk1^+^VE-Cadherin^+^CD41^*−*^ populations (right); (% cells in each quadrant indicated). (**d**) Number of cells with the indicated cell surface markers (VE-Cadherin^*−*^CD45^+^, VE-Cadherin^*−*^CD41^+^ and VE-Cadherin^+^) recovered after five-day culture on OP9 stroma. Mean cell number (per 25 × 10^3^ cultured cells) + SEM (error bars); three independent experiments; *p < 0.05 and NS: not significant (paired Student *t*-test).

Since our results show that EfnB2 is required for hematopoiesis from aorta and from ES-derived endothelial cell populations but not from YS or BL-CFC culture of ES/EBs, we tested if endogenous expression of EfnB2 in ES cells could enrich for precursors with a hemogenic potential. Unlike essential hematopoietic transcription factors that are intracellular, EfnB2 is a transmembrane protein with an extracellular domain recognized by antibodies and soluble Eph receptors^54^. Taking advantage of a specific EfnB2 antibody, we sorted EfnB2^+^ and EfnB2^*−*^ cell populations (Flk1^+^VE-Cadherin^*−*^CD41^*−*^ and Flk1^+^VE-Cadherin^+^CD41^*−*^) from Day 5 ES/EBs (schematic of experiment in Fig. 6a; sort purities Supplementary Fig. 8a). The percentage of EfnB2^+^ cells ranged from 7 to 20% of total live cells from Day 5 EBs (representative profiles, Supplementary Fig. 8a). Gene expression analysis showed that the EfnB2^+^ Flk1^+^VE-Cadherin^*−*^CD41^*−*^ sorted cells express higher levels of the hematopoietic transcription factors *Scl* (22-fold higher) and *Runx1* (30-fold higher) than the corresponding EfnB2^*−*^ population (Fig. 6b, blue bars). *Sox17*, which was previously used to enrich for hematopoietic progenitors in a reporter Sox17-mCherry ES cell line^34^, was also expressed at a higher level (3-fold) in the EfnB2^+^ than in the EfnB2^*−*^ Flk1^+^VE-Cadherin^*−*^CD41^*−*^ population (blue bars; Fig. 6b). In addition, EfnB2^+^ Flk1^+^VE-Cadherin^+^CD41^*−*^ sorted cells express somewhat higher levels of *Runx1* (3-fold higher) than the corresponding EfnB2^*−*^ population (Fig. 6b, red bars).

We examined if this EfnB2^+^ fraction has more hematopoietic potential than the EfnB2^*−*^ fraction. To this end, the sorted EfnB2^+^ and EfnB2^*−*^ (Flk1^+^VE-Cadherin^*−*^CD41^*−*^ and Flk1^+^VE-Cadherin^+^CD41^*−*^) cells (sorting purities, Supplementary Fig. 8a) were cultured onto OP9 stroma for five days and the output of VE-Cadherin^+^, CD41^+^ and CD45^+^ cells measured by flow cytometry (representative plots in Fig. 6c; cumulative results in Fig. 6d). We find that the EfnB2^+^ fraction of each of the sorted populations (VE-cadherin^+^ or VE-cadherin^*−*^) generates a significantly greater number of VE-cadherin^*−*^CD45^+^ and VE-cadherin^*−*^CD41^+^ hematopoietic cells compared to the EfnB2^*−*^ fraction (Fig. 6c,d). In contrast, we find that the EfnB2^+^ and the EfnB2^*−*^ fractions generate a similar number of VE-cadherin^+^ endothelial cells (Fig. 6c,d). Imaging confirmed that the EfnB2^+^ fractions give rise to more non-adherent hematopoietic-like cells than the EfnB2^*−*^ fraction (brightfield), but similar numbers of VE-Cadherin^+^ endothelial cell colonies (red) (Fig. 6e).

We also examined the output of hematopoietic cells from EfnB2^+^ and EfnB2^*−*^ cells (Flk1^+^VE-Cadherin^*−*^CD41^*−*^ and Flk1^+^VE-Cadherin^+^CD41^*−*^) from Day 6 ES/EB^40^ (Supplementary Fig. 8b,c). Confirming the results in Fig. 6, the EfnB2^+^ fraction from Day 6 ES/EB consistently generated a greater proportion of VE-Cadherin^*−*^CD45^+^ and VE-Cadherin^*−*^CD41^+^ cells than the EfnB2^*−*^ fraction (Supplementary Fig. 8b,c). Thus, in this system endogenous EfnB2 expression marks cells with the greatest hemogenic potential during ES differentiation.

## Discussion

Here we show that EfnB2 plays a previously unrecognized role as a principal regulator of hematopoiesis from the DA and ES cells by controlling the emergence of hemogenic endothelial cells. During vascular development, EfnB2 is essential for the proper sorting of arterial and venous-fated endothelium into distinct arterial and venous vascular beds. This activity is linked to activation of the EphB4 venous-restricted tyrosine kinase receptor. EfnB2 additionally controls VEGF/VEGF receptor-induced endothelial cell growth relying on receptor-induced PDZ-mediated “reverse” signaling^55^,^56^. We have recently shown that tyrosine phosphorylation-dependent EfnB2 signaling controls post-angiogenic vessel involution^54^. The current findings, demonstrating a selective functional role of EfnB2 in DA-derived hematopoiesis from endothelium, unveil yet another vascular function of EfnB2. Given that EfnB2 deficiency does not prevent formation of the DA, does not impair ES differentiation into endothelial cells and does not impair YS hematopoiesis, we interpret the current results as providing strong evidence that a requirement for EfnB2/EphB4 signaling distinguishes the hematopoietic program originating in the DA from other endothelial programs.

These observations are consistent with those from recent studies showing that Wnt-β-catenin signaling plays critical roles at multiple stages of definitive-type hematopoietic development but is not required for primitive-type hematopoiesis, despite both programs transition through an intermediate hemogenic endothelium *in vitro*^57^,^58^. The finding in both studies that distinct signaling pathways regulate DA-derived and YS-derived hematopoiesis provides further support for an independent emergence of the two hematopoietic programs^16^. Noteworthy, connections between Wnt and Eph/Efn signaling are well established in numerous tissues and morphogenic processes^59^,^60^, raising important questions on the relative contribution of these signaling pathways to regulation of DA-derived hematopoiesis. In the mouse, Wnt/β-catenin signaling was transiently required to generate HSC in the aorta-gonad-mesonephros (AGM) at E10.5 and E11.5^57^. In the human system, Wnt/β-catenin activation was proposed to target KDR^+^ precursors emerging from pluripotent stem cells on day 2–3 of differentiation^58^. Although differences of experimental systems prevent definitive conclusions, the current results point to an earlier functional role of EfnB2 compared to Wnt/β-catenin since the *EfnB2*-deficient DA is defective at producing hematopoietic cells *ex vivo* at E9.0–9.5, prior to the emergence of a putative sensitivity to Wnt/β-catenin signaling. In addition, *EfnB2* is expressed in differentiating mouse ES cells before *Flk1/KDR* and the silencing of EfnB2 at the outset of differentiation, prior to *Flk1/KDR* expression, impairs ES output of hematopoietic cells on OP9 stroma.

Our demonstration that EfnB2 spatiotemporally controls the emergence of hematopoiesis from the DA raises the interesting question of how this control is imposed, particularly in view of the broad endothelial expression of EfnB2. An involvement of EphB4/EfnB2 signaling is likely because the EphB4-deficient mice phenocopy the cardiovascular defects of EfnB2-deficient mice^25^, and EphB4 broadly regulates ES differentiation into mesodermal-derived tissues^61^. Cell-to-cell interaction is required for activation “*in trans*” of forward (EphB4-derived) or reverse (EphrinB2-derived) signaling. We show that EphB4/EfnB2 co-localize in selected neighboring endothelial cells of the emergent DA at about E8.5, providing an opportunity for bidirectional signaling. Consistent with this, previous studies have demonstrated that a subset of EphB4^+^/EfnB2^*−*^ endothelial cells transiently cohabit the developing DA at the 6–8 somite stage with EfnB2^+^/EphB4^*−*^ endothelial cells, prior to segregating to the cardinal vein^26^. This time-point closely coincides with the earliest identification of prospective hemogenic endothelial cells based on Runx1-regulated +23 enhancer-reporter tracking^62^. Thus, we propose that the temporally and spatially restricted residence of EphB4^+^ cells in the developing DA provides functional control of EfnB2 activation thereby initiating endothelial commitment to hematopoiesis. Consistent with a role of EfnB2 signaling in DA hematopoiesis, a mutant mouse line that expresses an *EphrinB2* lacking 66 amino acid residues of cytoplasmic tail resembles the EphrinB2-null line^63^, although this was not confirmed in a separately derived mouse line^64^.

Previous time-lapse imaging studies have documented the emergence of blood cells from ES-derived endothelium and the ventral endothelial wall of the DA^13^,^65^,^66^,^67^, but the timeline of endothelial cell commitment to hematopoiesis and other aspects of the process are incompletely defined. The absence of phenotypic features distinguishing hemogenic and non-hemogenic endothelium in the DA suggested a general competence of the DA endothelium to hematopoiesis, and the control for unfolding of the hematopoietic program attributed to Runx1-targeted signals from the subaortic mesenchyme^68^. Recent studies, however, support the view that the hemogenic endothelium may represent a distinct lineage of endothelial precursors, which can be positively distinguished from other endothelial cell components of the DA wall as early as two days before the emergence of hematopoiesis^62^. Other recent studies have characterized the hemogenic endothelial cells as the CD34^+^CD73^*−*^CD184^*−*^ fraction of human ES-derived embryoid bodies, segregating these cells from the CD34^+^CD73^hi/med^ CD184^*−*^ arterial and venous progenitors^37^. In showing that the silencing of EfnB2 at the outset of ES differentiation limits the development of a hemogenic endothelium and suggesting that hemogenic endothelial cells can be prospectively distinguished in the emergent DA at about E8.0-E8.5 (somite stage 6–8), our studies are in agreement with an early endothelial specification to hematopoiesis.

It will be of interest to assess the lineage relationship between the EfnB2^+^ precursors in the current study, the CD73^*−*^CD184^*−*^ hemogenic cells that have low EfnB2 mRNA identified in human ES-derived EB culture^37^, the 23GFP+ hemogenic endothelial cells identified in the developing DA before hematopoiesis is established^62^, the endothelial cells with adult-type hemogenic potential that bear a defined ratio of RUNX1 to SOX17 levels^69^, the kit^hi^CD41^+^CD16/32^+^ YS precursors^44^ and the recently identified lineage of YS endothelial cells that are specified before gastrulation^10^.

In conclusion, our results show that EfnB2 plays a previously unrecognized mechanistic role in controlling the commitment of endothelial precursors to the hematopoietic program and support a model in which this commitment begins early in the emergent DA when EfnB2 signaling is induced in selected endothelial cells from interaction with EphB4^+^ endothelial cells that transiently cohabit the emergent DA. These results have important implications for a mechanistic understanding of endothelial hematopoiesis, directing research to EfnB2-dependent signaling pathways and to earlier developmental events than previously considered. In turn, this impacts future research aimed at improving the generation of hematopoietic stem cells for effective regenerative therapies, which are not currently available.

## Methods

### Maintenance and differentiation of ES cells

The J1 mouse ES cell line was maintained in tissue culture vessels (6-well plates, Corning) pre-coated with 0.1% gelatin (Sigma G1890)/PBS in maintenance medium: Dulbecco’s Modified Eagle Medium (DMEM; Gibco 11965) supplemented with 15% pre-tested fetal calf serum (FCS; GE Healthcare/PAA Laboratories A15–101), 2% Leukemia-Inhibitory Factor (LIF)-conditioned medium (culture supernatant of CHO-LIF cell line, gift of G. Keller), 1% penicillin-streptomycin (Gibco 15140) and 1.5 × 10^*−*4^ M monothioglycerol (MTG; Sigma M6145). ES cells (used up to passage 7), were maintained at 37 °C with 5% CO_2_ and split (1:4–1:6 dilution) every 48 hours using 0.05% trypsin/EDTA (Gibco 25300; 3 minutes at 37 °C). Prior to differentiation, ES cells were cultured for 24 hr in IMDM (Gibco 31980) medium supplemented with the same components of maintenance medium under the same culture conditions. ES cells were differentiated into suspension embryoid bodies (EB) by culture (1 × 10^4^ cells mL^*−*1^) in differentiation medium: IMDM with 15% pre-tested FCS (GE Healthcare/PAA Laboratories A15–101), 2 mM L-glutamine (Gibco 25030), 200 μg mL^*−*1^ transferrin (Roche 10652202001), 0.5 mM ascorbic acid (Sigma A4544), 1% penicillin-streptomycin and 4.5 × 10^*−*4^ M MTG in 90 mm Petri dishes (Thermo 101 V/IRR). On Day 4 of differentiation, EBs in suspension were harvested, washed in PBS, trypsinized, strained through a 40 μM mesh (Greiner 542040) and “re-aggregated” by culture at 2.5 × 10^5^ cells mL^*−*1^ in IMDM differentiation medium (described above) with or without lentivirus^40^.

### Lentivirus production

Third-generation HIV1-based lentiviral short hairpin RNA (shRNA) constructs targeting EfnB2 were purchased from Sigma-Aldrich. The sequence (TRCN0000058427) CCGGCTGGTACTATACCCACAGATACTCGAGTATCTGTGGGTATAGTACCAGTTTTTG (targeting sequence underlined) was cloned by restriction digestion with the BamHI and KpnI enzymes and inserted into the plko.1-eGFP backbone replacing the puromycin resistance selection marker with enhanced green fluorescent protein (eGFP) under control of the PGK promoter. The same cloning was carried out for the empty vector plasmid to generate a PGK-eGFP control lentivirus^70^. The shRNA sequence (TRCN0000066493) CCGGCGGGTGTTACAGTAGCCTTATCTCGAGATAAGGCTACTGTAACACCCGTTTTTG (sequence underlined targets the 3′ UTR of mouse EfnB2) was used with the puromycin selection marker under control of the PGK promoter. Control plko.1-puromycin was used as the control. Virus-containing supernatant, collected 72 hours after transfection of 293 T cells (ATCC, no. CRL-3216) using Lipofectamine 2000 (Invitrogen 11668–500), was concentrated by ultracentrifugation (19,500 rpm for 2 hours and 20 minutes^70^). Each shRNA (puromycin and eGFP) virus and control virus was produced separately into 293 T cells and pooled after concentration. Infected cells were selected by sorting the GFP^+^ cells.

### Blast colony forming cells (BL-CFC) culture

Flk1^+^ cells sorted from differentiating Day 4 ES/EB were cultured (6 × 10^4^ cells mL^*−*1^) in IMDM containing 1% methylcellulose (Sigma M0512), 10% FCS (GE Healthcare/PAA A15–101), 2 mM L-glutamine, 1% penicillin-streptomycin, 300 μg mL^*−*1^ transferrin, 25 μg mL^*−*1^ ascorbic acid, 4.5 × 10^*−*4^ M MTG, 5 ng mL^*−*1^ mouse (m) VEGF (PeproTech 450–32), 5 ng mL^*−*1^ mIL-6 (PeproTech 216–16) and 100 ng mL^*−*1^ mKit Ligand (PeproTech 250–03 replacing 20% D4T conditioned medium^71^) on ultra-low adhesion 6-well plates (Corning 3471).

### OP9 co-culture

OP9 cells (gift of SE Jacobsen, Oxford Univ.) were maintained in αMEM (Gibco 12000) containing 20% FCS (Sigma F2442) and 1% penicillin-streptomycin by periodic (every two-three days) splitting 1 to 6 with 0.05% trypsin/EDTA. OP9 stromal cells were plated onto Collagen IV (50 μg mL^*−*1^, Sigma C5533 in PBS) -coated Ibidi μ-Slide I 0.8 luer channel slides (Ibidi 80196; 7,000 cells per channel slide) 24 hours prior to cell addition^40^. Sorted ES cells (2.5 × 10^4^), YS-derived cells (2.5 × 10^4^) or DA-derived cells (yield: 6.2–9.4 × 10^3^ cells from 10–10.5 DA) were cultured for 5 days (longer culture time is impaired by OP9 overgrowth) in the channel slide with 220 μL IMDM containing 10% FCS, 1% penicillin-streptomycin, 2 mM L-glutamine, 5 ng mL^*−*1^ mVEGF, 40 ng mL^*−*1^ mThrombopoietin (PeproTech 315–14) and 40 ng mL^*−*1^ mFlt3-Ligand (Fms-related tyrosine kinase 3 ligand; PeproTech 250–31 L)^40^.

### Methylcellulose colony-forming assays

For each assay, 50 × 10^3^ (ES-derived) or 25 × 10^4^ (YS-derived) cells mixed into 2 mL of Methocult3434 methylcellulose medium (Stem Cell Technologies 03434) were plated into each well (ultra-low adhesion 6 well plate, Corning 3471). Alternatively, 10.4 × 10^3^  ES-derived cells mixed into 1 mL of Methocult3434 media were plated in each well (ultra-low adhesion 24 well plate; Corning 3473). Colonies were scored by an observer without knowledge of the experimental design between Days 5–12 of differentiation. Colony counts were normalized to 50 × 10^3^ input cells.

### Flow cytometry and cell sorting

Cells were dissociated using 0.05% trypsin/EDTA (3 minutes; 37 °C), strained through a 40 μM mesh and incubated at 37 °C for 45 minutes to allow reconstitution of surface proteins sensitive to trypsin digestion. Cell staining (carried out on ice; 45 minutes with up to 5.0 × 10^6^ cells mL^*−*1^ unless otherwise noted) utilized the following antibodies: PE-labeled Flk1 (Avas12a1; eBioscience 12–5821–83; 1:100; 20 minutes), biotinylated-EfnB2 (R&D BAF496; 20 μg mL^*−*1^), e-Fluor 450-labelled CD41 (MWReg30; eBioscience 48–0411–82; 1:150), PE-labeled VE-Cadherin (BV13; eBioscience 12–1441–82; 1:50), PE-Cy7-labelled c-kit (2B8; Biolegend 105813; 1:1000), PE-Cy5-labelled CD45 (30-F11; Biolegend 103109; 1:100), APC-labelled CD11b (BD Biosciences 553312; 1:100), APC-Cy7-labelled Ly6G (BD Biosciences, 560600, 1:100), PE-Cy5-labelled Ter119 (Biolegend 116210, 1:100) and FITC-labelled F4/80 (eBiosciences 11–4801–82, 1:100). Streptavidin-PE-Cy5 (BD Bioscience 554062; 1:400; 5 minutes on ice) was used to visualize the biotinylated-EfnB2 antibody. Cell viability was evaluated with propidium iodide (1:50; Life Technologies P3566) or DAPI (50 μg mL^*−*1^; Invitrogen D3571). Cells were sorted with a FACSAria IIu (BD) and results were analyzed on a LSR Fortessa (BD). Sample acquisition was carried out with FACSDiva software (BD; Version 8.0.1) and results were analyzed and displayed using FlowJo software (FlowJo; Version 7.6.5).

### Live cell staining and imaging

Culture medium in Ibidi μ-Slide I channel slides was carefully replaced with 10% FCS/PBS buffer containing antibodies using 1 or 3 mL luer lock syringes to minimize non-adherent cell loss. Cells were stained for 30 minutes at 37 °C with the following antibodies: eFluor45-labelled CD41 (MWReg30; eBioscience 48–0411–82; 1:150), PE-labeled VE-Cadherin (BV13; eBioscience 12–1441–82; 1:50) and Alexa-488 labeled CD45 (30-F11; Biolegend 103121; 1:100). The antibody-containing buffer was carefully replaced with 10% FCS/PBS buffer using luer lock syringes before imaging using an Olympus IX51 inverted scope (Olympus) and image acquisition using a QImaging cooled mono 14-bit camera (Model 01-EXi-AQA-R-F-M-14-C) and IPLab software (BioVision Tech; Version 4.08); acquisition settings were kept identical for all samples. Images were selectively adjusted for brightness and contrast using Photoshop CC (Adobe; Version 14.0).

### Mice, timed mating and embryo collection

The previously characterized EfnB2^lacZ/+^ mice (gift of Drs. S. Raft and Y. Mukouyama, NIH, Bethesda, MD)^22^ were used in compliance with protocols approved by the NCI IACUC committee; all mouse experiments were carried out in “accordance” with the approved guidelines. Pregnancies were timed at E0.5 based on the presence of a vaginal plug. Pregnant mothers were euthanized for embryo dissection at the appropriate developmental stage. Only live embryos with beating hearts were dissected and somites were counted for embryo staging. Aortas, dissected as described^27^ into cold 10% FCS/PBS, were dissociated with 0.12% Collagenase I (Sigma C1639) for 30–40 minutes at 37 °C (with pipetting every 10 minutes) until no visible tissue fragments remained. Trypsin activity was blocked at the same time to ensure uniformity. Somite tissue contamination of dissected aortas was evaluated by measuring MyoD expression levels by qPCR. Dissected DAs had a relative (% GAPDH) MyoD expression <0.001 of GAPDH at E9.0–9.5 and <0.01% at E10.0–10.5. The YSs were dissected from the embryo into cold 10% FCS/PBS and dissociated with 0.24% Collagenase I (50–70 minutes at 37 °C with pipetting every 10 minutes) until no/minimal visible tissue fragments remained (YS from each litter was quenched at the same time). Whole embryos used for PLA were trimmed of head and one side of the body wall for optimal imaging. Embryo remnants were frozen for genotyping using primers listed by Jackson Labs (Stock Number 006039).

### Tissue culture *ex vivo*

For culture of aortas, OP9 stromal cells were plated onto Collagen IV-coated Ibidi channel slides to generate a monolayer over 24 hours, as described above. Each dissociated aorta (1 embryo equivalent) was cultured for five days in a single Ibidi channel slide in 220 μL IMDM containing 10% FCS (GE Healthcare/PAA A15–101), 1% penicillin-streptomycin-neomycin (Gibco 15640), 50 μg mL^*−*1^ ascorbic acid, 300 μg mL^*−*1^ transferrin, 1.5 × 10^*−*4^ M MTG, 1 ng mL^*−*1^ mIL-7 (PeproTech 217–17), 5 ng mL^*−*1^ mIL-11 (PeproTech 220–11), 100 ng mL^*−*1^ mKit Ligand, 5 ng mL^*−*1^ mVEGF, 40 ng mL^*−*1^ mThrombopoietin, and 40 ng mL^*−*1^ mFlt3Ligand. For culture of YS, 0.2 embryo equivalent of dissociated E9–9.5 YS was cultured into a single well (6-well plate, Corning 3471) in 2 mL MethoCult3434 methylcellulose medium with 1% penicillin-streptomycin-neomycin. All younger YS (1 embryo equivalent) were cultured into a single well (24 well plate, Corning 3473) in 1 mL of MethoCult3434 medium with 1% penicillin-streptomycin-neomycin. Colonies were counted by observer without knowledge of the experiments between Day 9–12 of culture and normalized to 1 embryo equivalent.

### Gene expression by qPCR

Up to 5 × 10^5^ cells were sorted directly into RNeasy Micro kit lysis solution (Qiagen 74004). YS and aorta were dissected directly into RNeasy Micro kit lysis solution. RNA was extracted following kit instructions, including the step for on-column DNase digestion. cDNA was synthesized using a High-Capacity cDNA Reverse Transcription kit (Applied Biosystems 4368814). SYBR Green reactions were performed according to kit instructions (Roche 11282400). Taqman reaction (Applied Biosystems 4304437) primers are listed in Supplemental Table 2. qPCR was run on a 7900HT Fast RT PCR System (Applied Biosystems) for 40 cycles with an annealing temperature of 60 °C. GAPDH was used as an endogenous reference control. Quantitation of gene expression results was performed using the ΔΔC_T_ method (ΔΔC_T =_ ΔC_T_ test sample − ΔC_T_ calibrator sample), as detailed: http://www3.appliedbiosystems.com/cms/groups/mcb_support/documents/generaldocuments/cms_042380.pdf. The results are expressed as relative mRNA levels.

### Erythroid cells immunostaining

After fixation in 4% PFA/PBS (4 h at room temperature, RT), samples were permeabilized/blocked in blocking buffer (1% Triton/2.5% BSA/10% glycerol/10 mM glycine/TBS, pH 7.4; 1 hr) and treated with Streptavidin/Biotin reagents (Vector, SP-2002; 1 hr each) and Fc Receptor blocker (Innovex Bioscience, NB309–15). Staining strategy: samples were first stained with rabbit antibodies to fetal βH1 globin followed by AlexaFluor488-Zenon goat Fab-antirabbit IgG (Fc), Fab-antirabbit IgG blocking and re-fixation, and subsequently stained with rabbit antibodies to adult Beta-t globin followed by AlexaFluor594 goat anti-rabbit IgG. Specifically, after washing with blocking buffer, YSs were incubated (4 °C 18 hr) with anti-HBG2 rabbit antibody (1:25 dilution, LifeSpan BioScience, LS-C344603; recognizes epitope aa101–133 of human Gamma-2-globin and mouse βH1 globin) or rabbit IgG (1 mg/ml). After washing with blocking buffer, re-fixation with 4% PFA/PBS (20 min, RT), samples were incubated (2 hr, RT) with AlexaFluor488-Zenon goat Fab-anti-rabbit IgG (Fc) (Life Technologies, S32357). After washing, samples were incubated with unlabeled Fab fragment of anti-rabbit IgG (Jackson Immunoresearch, 711–007–0032 hr, RT) and re-fixed (4%PFA/PBS 20 min at RT). After washing, samples were incubated (4 °C, 18 hr) with anti-HBB antibody (Proteintech, 16216-1-AP; human gene ID3034, mouse ortholog Hbb-bt/“hemoglobin, beta adult t-chain”/Beta-t) or rabbit IgG (1 mg/ml). After washing, the samples were incubated (2 hr, RT) with AlexaFluor594 donkey anti-rabbit IgG (H + L chains, Life Technologies, A11056). After washing, samples were re-fixed (4% PFA/PBS 20 min RT). Samples were clarified (RapiClear solution/50% water, 2 hr RT) and YSs flattened onto coverslips under microscopy. Images were obtained with Carl Zeiss LSM780 (Carl Zeiss). Mean fluorescence intensity/cell was measured by ImageJ.

### Proximity ligation assay

PLA was used to visualize proximity co-localization (<40 nm) of EphrinB2 + EphB4 in mouse embryos (E8.5) after removal of the head and one side of the body wall, as described^72^. After dehydration with ethanol, wash (1% Triton X-100/PBS) and antigen retrieval (Uni-Trieve solution (Innova Bioscience), the embryo was first immunostained for CD31 by blocking (1 hr, at room temperature) with 0.2% BSA/1% milk/0.4% Triton X-100/TBS (BSA/TBS-MT); incubation (2 days at 4 °C) with anti-mouse CD31 rat mAb (1:20, clone SZ31, HistoBiotech, code DIA-310); wash (1 hr + 18 hr at 4 °C); incubation with AlexaFluor 488-conjugated Fab anti-rat IgG (5 μg/ml, 24 hr at 4 °C; Jackson ImmunoResearch); wash (1 hr + 18 hr at 4 °C); fixation with 4% PFA/PBS (1 hr at room temperature); and wash (1 hr + 1 hr at room temperature). For PLA, anti-EphB4 rat mAb (ab73259, Abcam) was conjugated with DNA probe, Duolink PLUS using Duolink Duolink® *in Situ* Probemaker PLUS kit (DUO92009, Olink). After blocking (1 hr at room temperature) with 0.2% BSA/10 mM glycine/5 mM EDTA/0.4% Triton X-100/TBS, the embryo was incubated (2 days at 4 °C) with EphrinB2 rabbit mAb (ab150411, Abcam) and Duolink PLUS-conjugated EphB4 rat mAb. After washing with Duolink Wash Buffer A (3 washes, 1 hr each), incubation with PLA probe anti-rabbit MINUS (92005–0030, Olink; 2 days at 4 °C), wash with Duolink wash buffer A (3 times, 1 hr each), incubation with ligation solution (18 hr at 37 °C), wash with Duolink wash buffer B (1× twice, 1 hr each; followed by 0.01× twice 1 min; followed by 1× once, 1 hr), the embryo was fixed with 4% PFA/PBS (1 hr at room temperature) and then washed (1 hr at room temperature) with 10 mM glycine/TE containing 0.5 mg/ml Hoechst 33342. The tissue was clarified by 24 hr incubation at 37 °C in RapiClear working solution (1:2 in water) and soaked into RapiClear original solution (18 hr at 4 °C). Images (1400 images) were obtained with Carl Zeiss LSM780 (Carl Zeiss). For 3D reconstruction, z-stack images were obtained from region of dorsal aorta with 0.496 μm thickness (150 images per field x 3 fields) using Carl Zeiss LSM780. The DA region was reconstructed as 3D object from the z-stack images using Zen software, blue edition (Carl Zeiss), and then images of longitudinal and cross-section slices of DA were generated from the reconstructed 3D embryos.

### Statistical analysis

All results from qPCR and colony counts, compiled and graphed either in Microsoft Excel or GraphPad Prism6 (Manufacturer), are expressed as mean ± standard error of the mean (SEM) unless otherwise noted. Statistical significance of differences between two groups was calculated using two-tailed Student’s t-test in GraphPad Prism6. Paired testing was used for analyses of *in vitro* experiments and unpaired testing was used for analysis of *ex vivo* experiments. The results are provided as P values, where P < 0.05 is considered statistically significant.

## Additional Information

**How to cite this article**: Chen, I.-I. *et al*. EphrinB2 regulates the emergence of a hemogenic endothelium from the aorta. *Sci. Rep.* **6**, 27195; doi: 10.1038/srep27195 (2016).

## Supplementary Material

Supplementary Information
