# Supplementary material for: EphrinB2 regulates the emergence of a hemogenic endothelium from the aorta
Source: Sci Rep. 2016 Jun 2;6:27195. doi: 10.1038/srep27195 (PMC4890174; doi:10.1038/srep27195)
Supplement: Supplementary Information [file srep27195-s1.pdf]

Supplementary Information

EphrinB2 regulates the emergence of a hemogenic endothelium from the aorta

Inn-Inn Chen, Arianna Caprioli, Hidetaka Ohnuki, Hyeongyl Kwak, Catherine Porcher & Giovanna Tosato

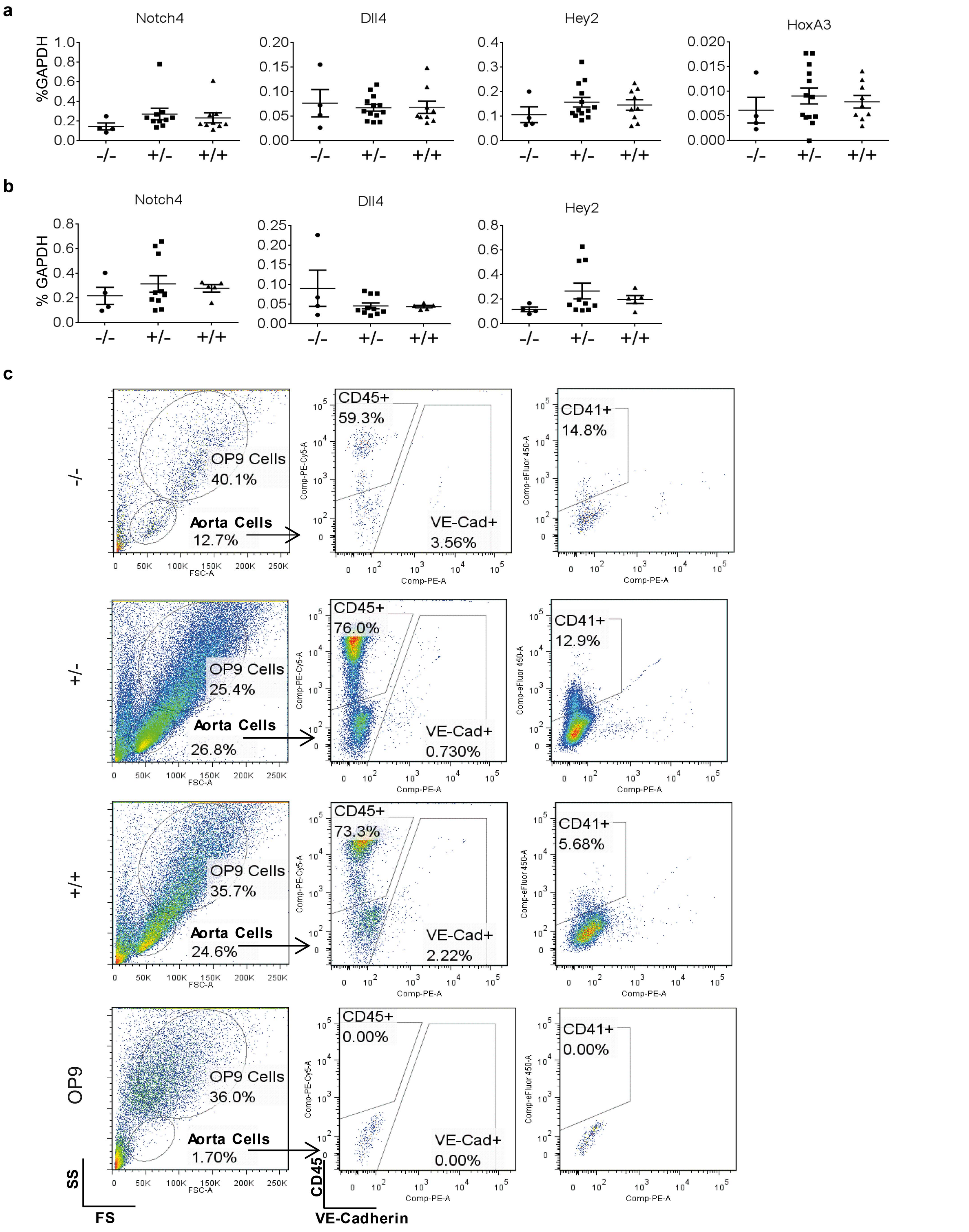

Supplementary Fig. 1 related to Fig. 1| Characterization of ***EfnB2*** $^{-/-}$ **DA.** (**a**,**b**) Relative mRNA levels (% GAPDH) were measured by qPCR in aortas resected from *EfnB2* $^{-/-}$ (n=4), *EfnB2* $^{+/-}$ (n=13) and *EfnB2* $^{+/+}$ (n=5-7) littermate embryos (4 litters) at E9.0-9.5 (**a**, 18-28 somite stage) and E10.0-10.5 (**b**, 20-36 somite stage); individual data points (dots, squares and triangles) and means (horizontal lines) ± SEM (error bars) are shown. P values from unpaired Student t-test; *P <0.05. (c) Representative flow cytometry analysis of cells recovered from 5-day culture of single-cell suspended *EfnB2* $^{-/-}$, *EfnB2* $^{+/-}$, and *EfnB2* $^{+/+}$ aortas at E9.0-9.5.

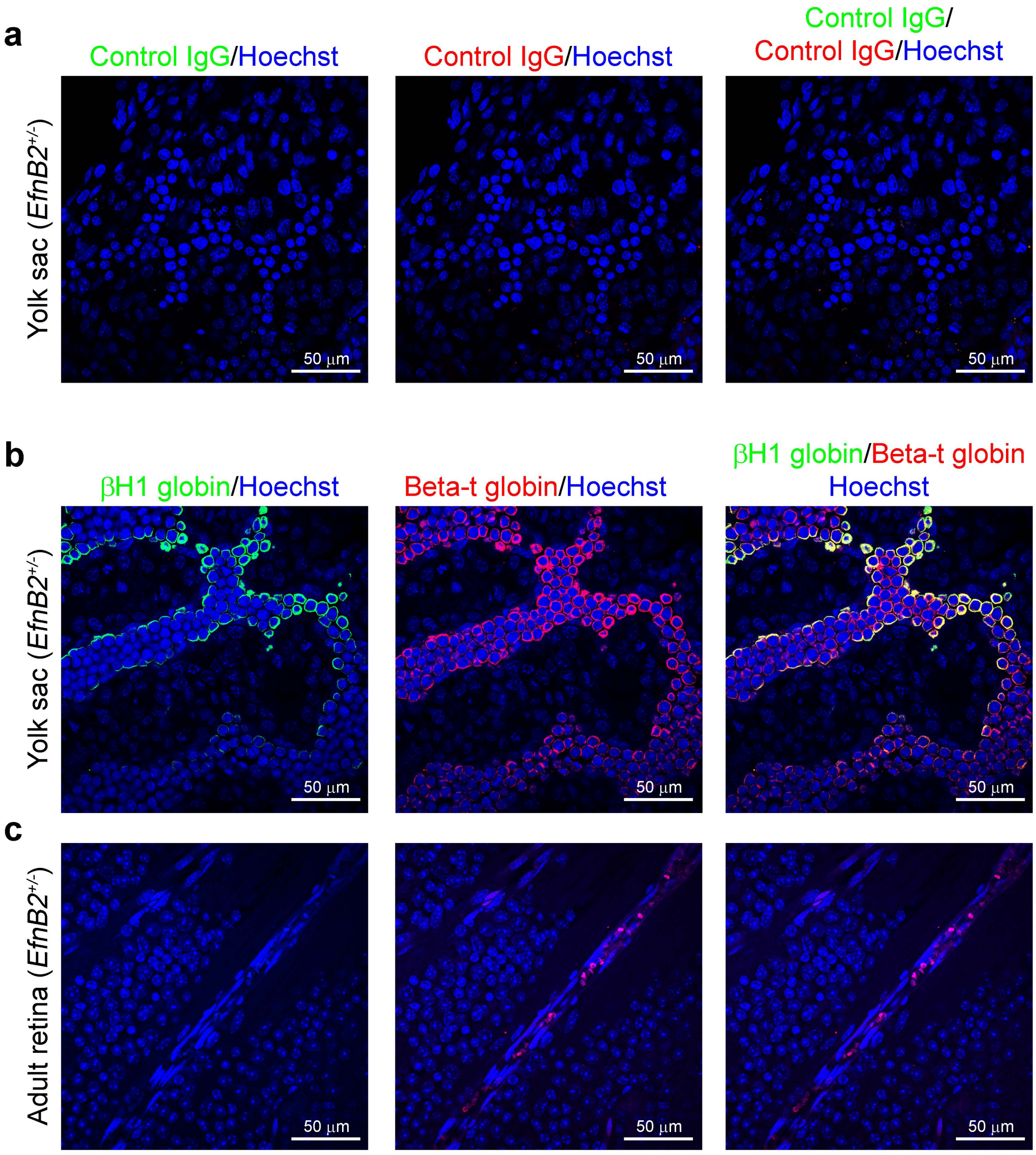

Supplementary Fig. 2 related to Fig. 2 | Immunofluorescent detection of fetal and adult globin proteins in YS erythroid cells. (a) Specificity controls for immunostaining with rabbit antibodies to adult Beta-t and fetal βH1 globins. Rabbit IgG replaced primary rabbit antibody in the staining protocol. **(b)** Specific detection of fetal βH1 globin (green) and adult Beta-t globin (red) in subsets of nucleated (Hoechst^{+}, blue) cells within YS tissue from an *EfnB2*$^{+/-}$ embryo at E9.5 (parallel staining with the specificity controls shown in a). Yellow color reflects detection of fetal βH1 globin (green) and adult Beta-t globin (red) within individual nucleated cells. **(c)** Immunostaining of adult mouse retina from an *EfnB2+/-* mouse shows specific detection of adult Beta-t globin (red) but not fetal βH1 globin (green) in cells within a retinal vessel; nuclei are Hoechst^{+} (blue).

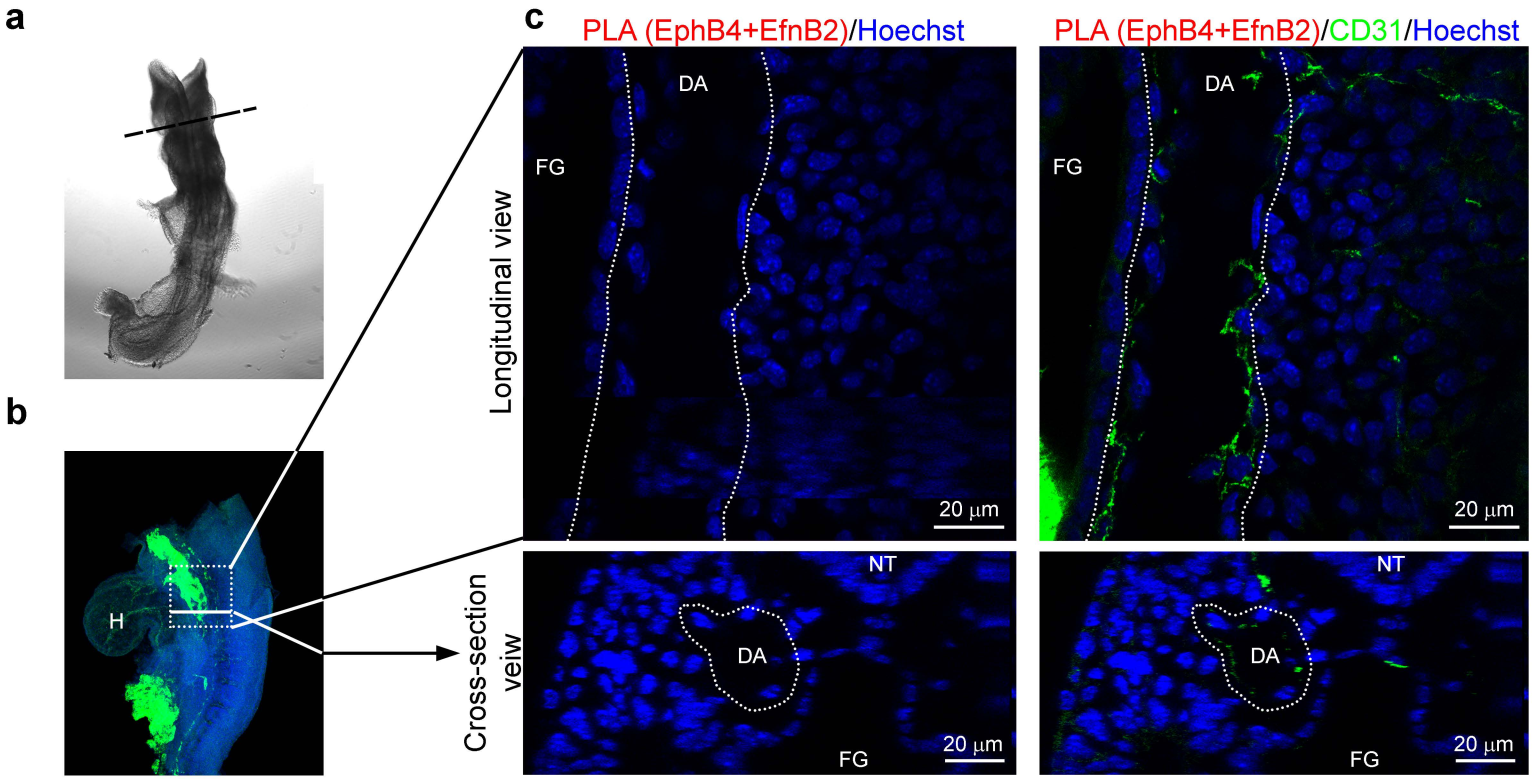

Supplementary Fig. 3 related to Fig. 3 | PLA control for Figure 5 in littermate *EfnB2*$^{-/-}$ embryo at E8.5. (a) Dissected embryo visualized by bright-field imaging prior to processing. The dotted line indicates the location of head removal from embryo in test sample. **(b)** Clarified embryo (E8.5) after removal of the head and side wall; CD31 immunostaining (green); Hoechst staining (blue); and PLA (pink, not detected). The dotted box limits the magnified area in the longitudinal view in c, upper panels; the horizontal line indicates the location of the cross-section magnified in c, lower panels. **(c)** CD31 immunostaining marks the *EfnB2*$^{-/-}$ DA endothelium; PLA (EphrinB2+EphB4) signal is not detected. The DA is outlined by the dotted line. FG: foregut; NT: neural tube.

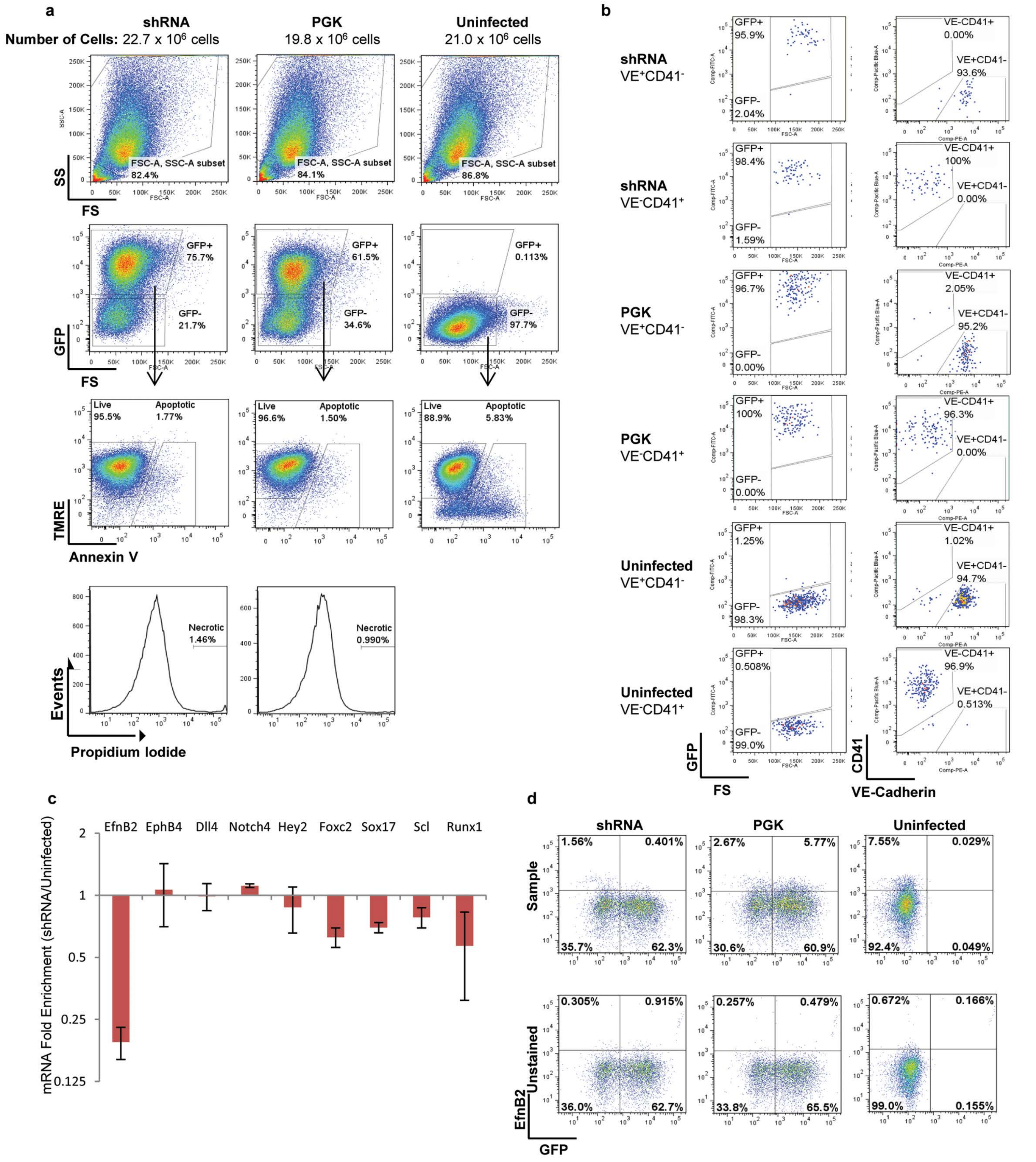

Supplementary Fig. 4 related to Fig. 4| Effects of EfnB2 silencing on ES-derived hemogenic endothelium. **(a)** Representative flow cytometry analysis of ES/EB recovered 48 hours after shRNA/PGK/no infection at differentiation Day 6. Cells were stained with TMRE (tetramethylrhodamine, ethyl ester, perchlorate), Annexin V and propidium iodide. Cell numbers at the top are the number of cells recovered from culture (three 10cm-dishes) of each ES/EB group on Day 6 of differentiation. (**b**) Purity of VE-Cadherin$^+$CD41$^-$ and VE-cadherin$^-$CD41$^+$ cell populations after sorting from shRNA/PGK/Uninfected ES/EB at Day 6 of differentiation. (**c**) Fold mRNA enrichment in GFP$^+$VE$^+$CD41$^-$ cells (shRNA versus Uninfected) at Day 6 differentiation. Fold change: ratio of GAPDH-normalized value in the shRNA sample/the corresponding PGK value. The bar graph reflects means ± SEM (error bars); log2 scale; three independent experiments. (**d**) Cell surface EfnB2 is reduced in Day 6 ES/EB after EfnB2 silencing. Flow cytometry: top row: sample; bottom row: unstained control.

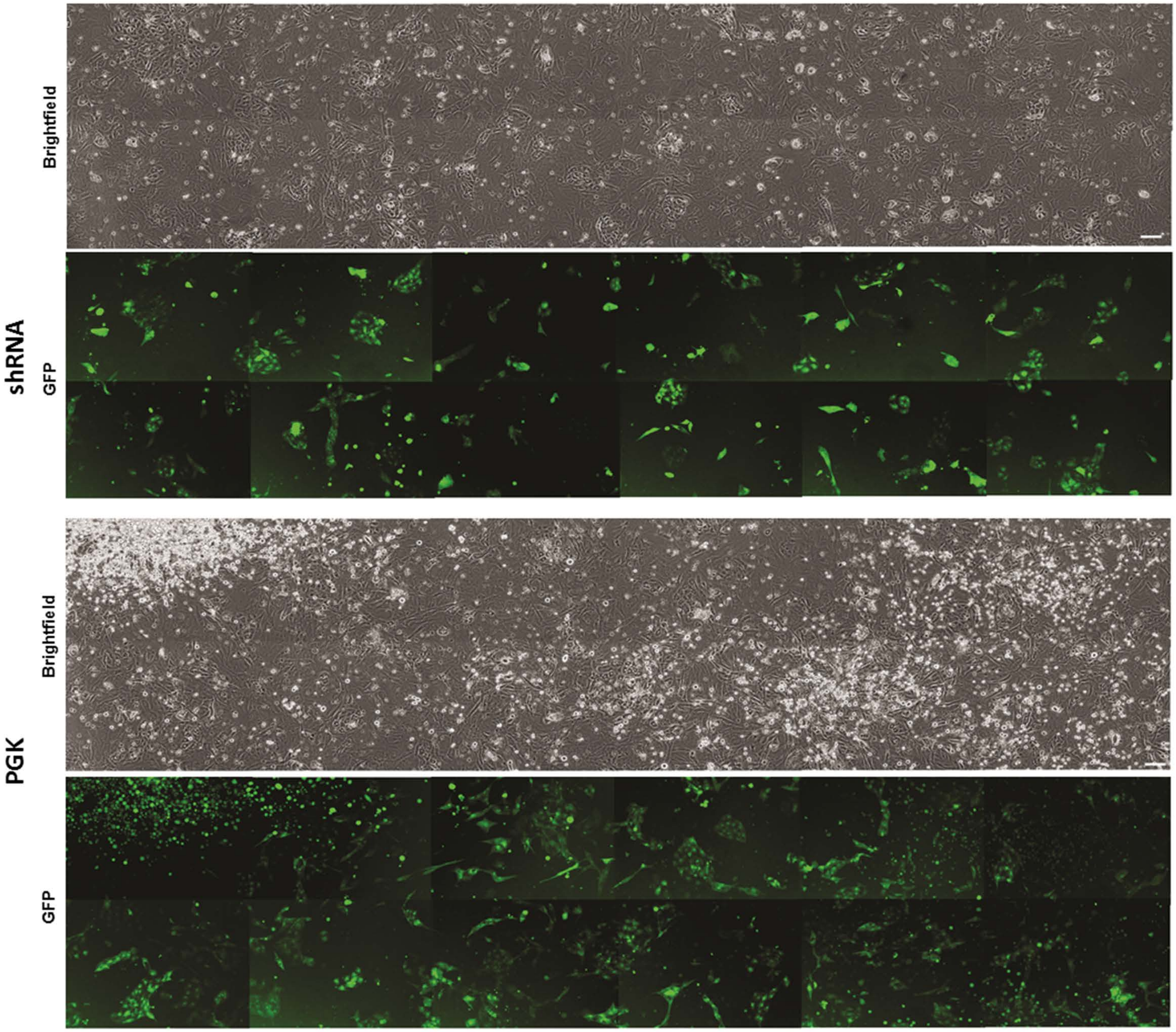

Supplementary Fig. 5 related to Fig. 4 | EfnB2 silencing impairs hematopoiesis from ES-derived endothelium but not the growth/survival of ES-derived endothelium. Tiled images of ES cells on Day 5 co-culture with OP9 stroma.. On Day 6 of differentiation, ES-derived GFP^{+}VE-Cadherin^{+}CD41^{-} cells were sorted (after infection on Day 4 with shRNA/PGK), and co-cultured with OP9 cells. Brightfield images show hematopoietic-type colonies in PGK-infected cultures, which are rare in shRNA cultures. Fluorescent images (GFP) show similar adherent endothelial-type colonies in shRNA and PGK-infected cultures (Scale bar: 100 μM).

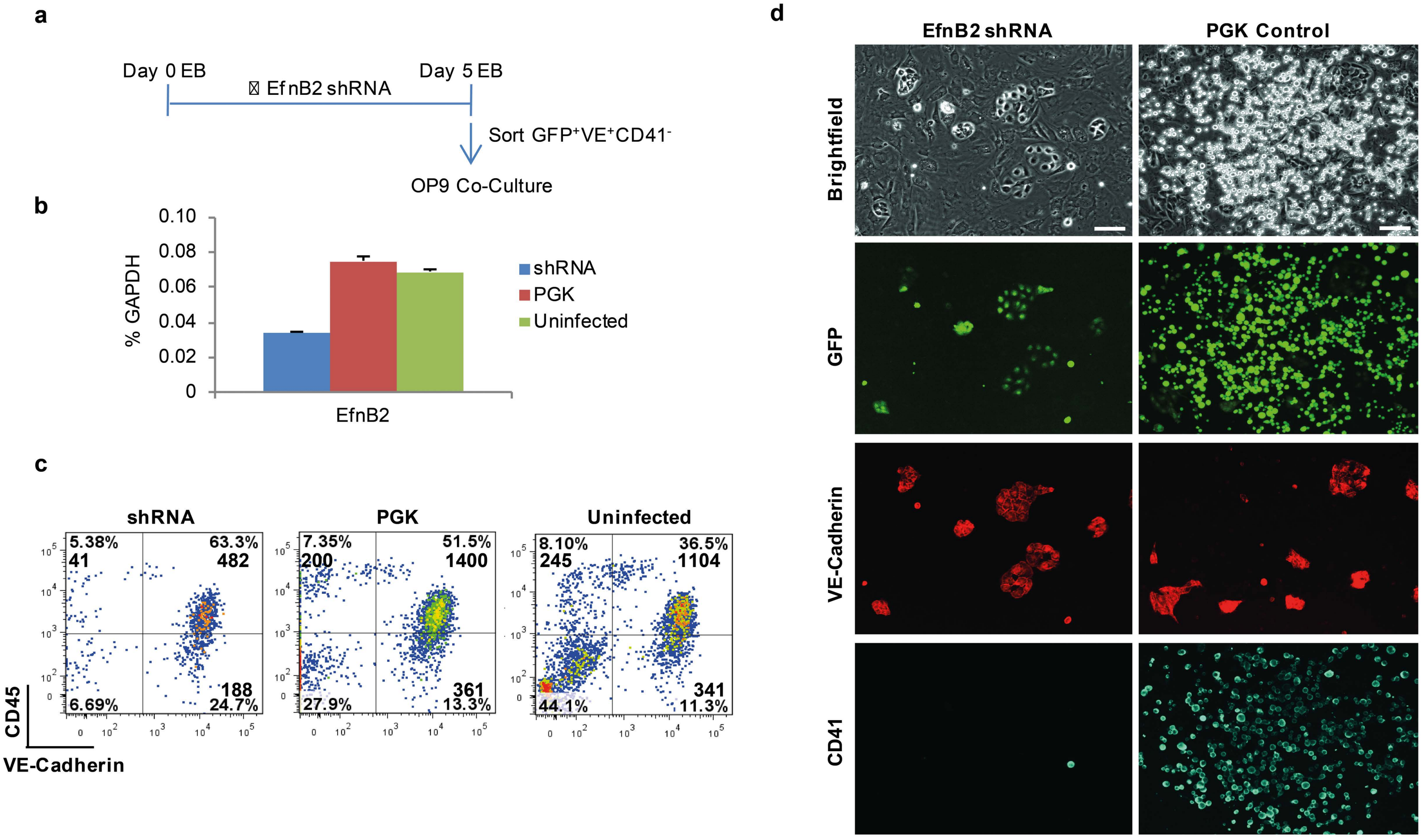

Supplementary Fig. 6 related to Fig. 4| Effects of EfnB2 silencing at the outset of ES/EB differentiation. (a) Schematic of the experiment. ES cells were infected with EfnB2 shRNA/PGK at Day 0 of differentiation or were left uninfected. On Day 5 of ES/EB differentiation, $GFP^+Flk1^+VE\text{-}Cadherin^+CD41^-$ cells were sorted from infected cultures and $Flk1^+VE\text{-}Cadherin^+CD41^-$ cells were sorted from uninfected cultures. The sorted cells were co-cultured with OP9 stroma for five days. **(b)** Reduced relative *EfnB2* mRNA in $Flk1^+VE\text{-}Cadherin^+CD41^-$ cells sorted on Day 5 of differentiation from shRNA-infected cultures compared to PGK-infected and uninfected cultures. The bar graph shows the means ± SEM (error bars); three independent experiments. **(c)** Representative FACS profiles of $GFP^+Flk1^+VE\text{-}Cadherin^+CD41^-$ and $Flk1^+VE\text{-}Cadherin^+CD41^-$ (uninfected cultures) recovered at the end of 5-day OP9 co-culture. Percent cells in each quadrant is listed; the event count in each quadrant is also listed. (GFP^+ gate for shRNA/PGK plots). **(d)** Immunofluorescent detection of VE-cadherin (red), CD41 (blue) and GFP (greeen) after 5-day OP9 co-culture of sorted $GFP^+VE\text{-}Cadherin^+CD41^-$ cells; corresponding brightfield images in the top quadrants. Representative results. Scale bars: 10 μm.

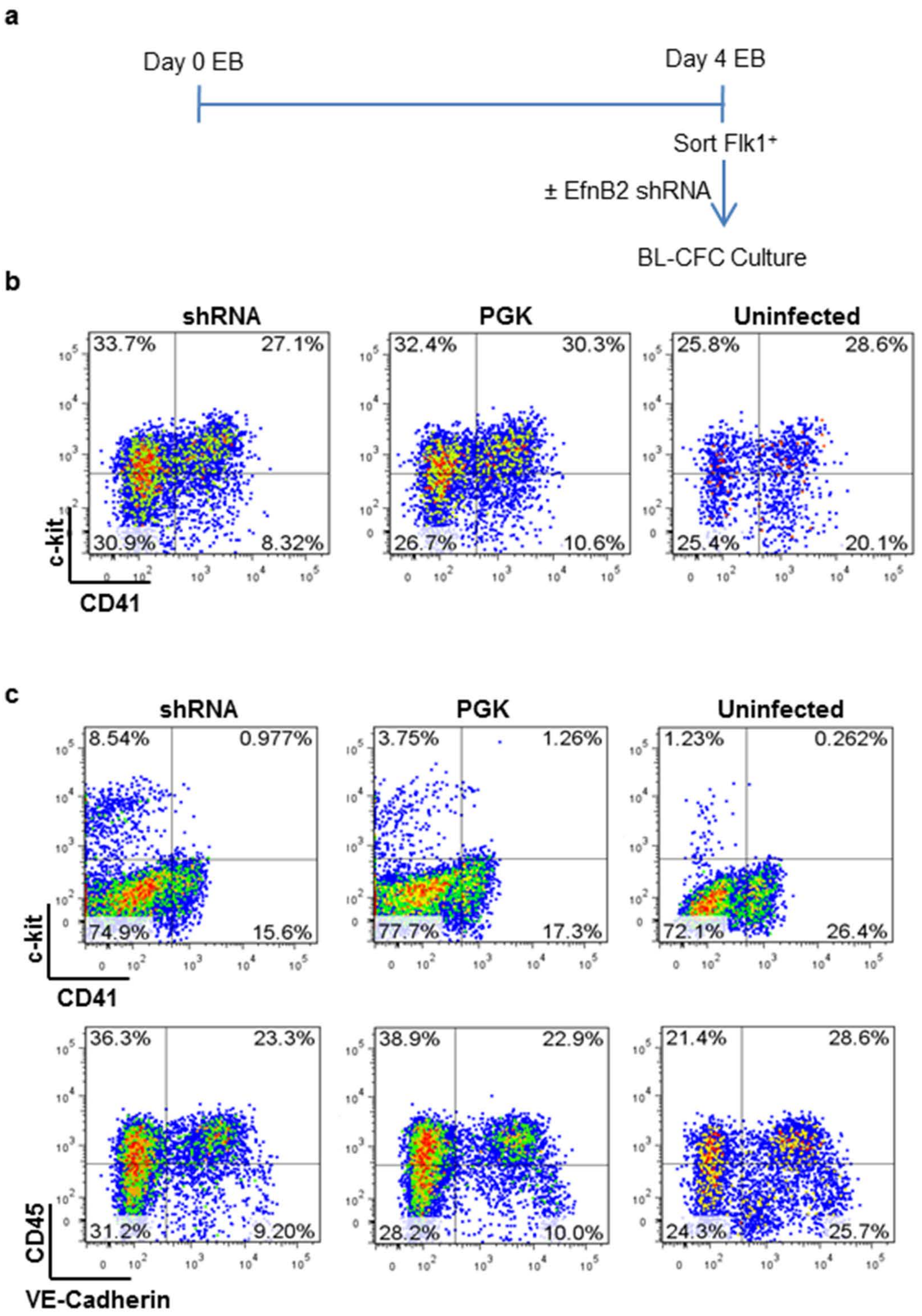

Supplementary Fig. 7 related to Fig. 5 | EfnB2 silencing in ES cells does not reduce hematopoietic differentiation in Blast Colony Forming Cell culture. **(a)** Schematic of experiment. $Flk1^+$ cells sorted from differentiation culture of ES/ EB on Day 4 were transferred to BL-CFC culture in the presence of shRNA/PGK lentivirus or were left uninfected. **(b,c)** Representative FACS plots of cells recovered at the end of BL-CFC culture for two days **(b)** and four days **(c)**. (GFP^+ gate applied to shRNA/PGK infected cells; representative profiles from three independent experiments).

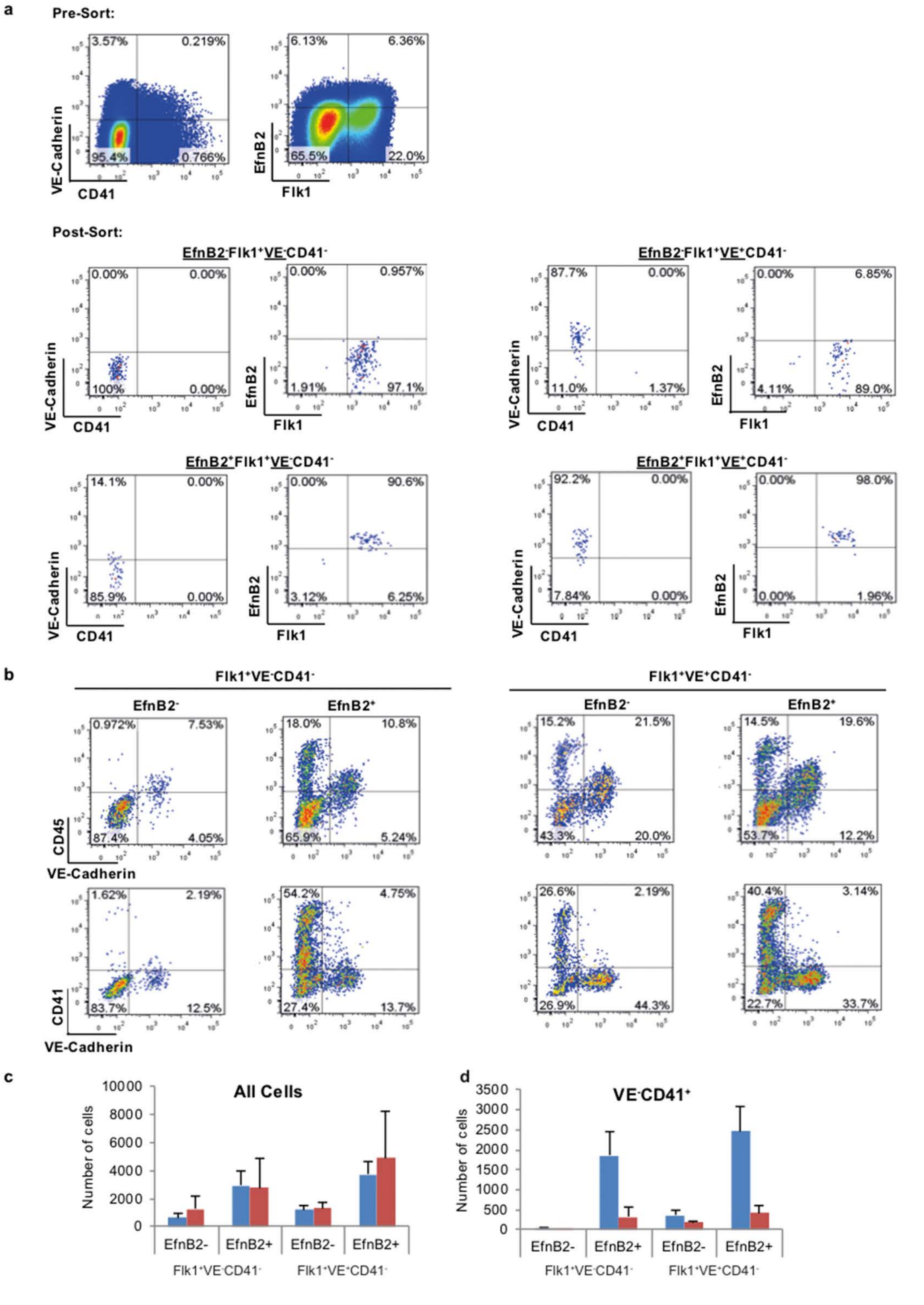

Supplementary Fig. 8 related to Fig. 6| EfnB2 sort purities and relationship between endogenous EfnB2 and ES hematopoietic differentiation. Representative FACS profiles of ES/EBs at Day 5 of differentiation **(a)** prior to sorting and **(b)** after sorting. **(c)** Representative FACS profiles of cells recovered from 5-day OP9 co-culture of EfnB2^{+} and EfnB2^{-}Flk1^{+}VE^{-}CD41^{-} and Flk1^{+}VE^{+} CD41^{-} from Day 6 EBs. **(d)** Total number of cells and total number of VE-Cadherin^{-}CD41^{+} cells recovered from 5-day OP9 co-culture of EfnB2^{+} and EfnB2^{-} Flk1^{+}VE^{-}CD41^{-} and Flk1^{+}VE^{+}CD41^{-} from Day 5 ES/EBs (blue bars) and Day 6 ES/ EBs (red bars). (Mean + SEM recoveries from per 25,000 seeded ES cells; three independent experiments).

	Exp1	Exp2	Exp3	Exp4	Avg	SEM
shRNA	8.3	10.6	13.7	21	13.40	2.76
PGK	12.7	12.2	20.7	57.8	25.85	10.83
Uninfected	43.4	13.2	28.2	44.5	32.33	7.38

Supplemental Table 1 related to Fig. 5| Recovery of Flk1+ cells from ES cells during differentiation. Percentages, averages (Avg) and SEM $Flk1^+$ cells recovered in four independent experiments from uninfected and infected (shRNA and PGK) ES/EB cultures (GFP^+ gate for shRNA/ PGK samples). Reduced $Flk1^+$ percentages in the shRNA versus PGK/ Uninfected controls is insignificant (paired Student t-test $p<0.05$).

	Forward Primer	Reverse Primer
GAPDH	TGT AGA CCA TGT AGT TGA GGT CA	AGG TCG GTG TGA ACG GAT TTG
VE-Cadherin	CCA CTG CTT TGG GAG CCT T	GGC AGG TAG CAT GTT GGG G
Foxc2	AAC CCA ACA GCA AAC TTT CCC	GCG TAG CTC GAT AGG GCA G
Sox17	TCC ATG AGG TGA CAT GCT GAG GTT	AGC TCC AGA AAC TGC AGA CCA GAA
Hey2	AAG CGC CCT TGT GAG GAA AC	GGT AGT TGT CGG TGA ATT GGA C
Notch4	CTC TTG CCA CTC AAT TTC CCT	TTG CAG AGT TGG GTA TCC CTG
EphB4	TGGCTGATCACGAACTTGACCTAC	AGG CAG GAC TCG TCT CCT ATT T
Scl	CAC TAG GCA GTG GGT TCT TTG	GGT GTG AGG ACC ATC AGA AAT CT
Runx1	GCA ACT TGT GGC GGA TTT GTA	GCA GGC AAC GAT GAA AAC TAC T

Supplemental Table 2 related to Methods | Sequences of qPCR primers used for mRNA quantitation. Taqman primers were used for measuring EfnB2, Dll4 and HoxA3 mRNAs.
